# Economic evaluations of preventive interventions for self-harm and suicide: a systematic review

**DOI:** 10.1017/S0033291726104814

**Published:** 2026-06-17

**Authors:** Long Khanh-Dao Le, Dai Quy Le, Phuong Hong Le, Eng Joo Tan, Amanda Neil, Karl Andriessen, Jane Pirkis, Cathrine Mihalopoulos, Lennart Reifels

**Affiliations:** 1 Health Economics Group, Monash University, Melbourne, Australia; 2 Menzies Institute for Medical Research, University of Tasmania, Hobart, Australia; 3 ALIVE National Centre for Mental Health Research Translation, University of Tasmania, Hobart, Australia; 4 Centre for Mental Health and Community Wellbeing, Melbourne School of Population and Global Health, University of Melbourne, Melbourne, Australia

**Keywords:** cost-effectiveness, economic evaluation, prevention, self-harm, suicide, systematic review

## Abstract

Self-harm and suicide are major public health concerns and leading causes of mortality worldwide, highlighting a pressing need for policymakers to identify and implement cost-effective interventions. This systematic review (PROSPERO registration #CRD42023460339) followed the PRISMA guidelines and aimed to synthesize the available cost-effectiveness evidence for the prevention of self-harm and suicide. Systematic searches were performed in MEDLINE, Embase, PsycINFO, CINAHL, Econlit, and ProQuest to identify full economic evaluations and return-on-investment studies on preventive interventions for self-harm and suicide published up to January 15, 2026. Methodological quality was assessed using Drummond’s 10-item checklist, and findings were synthesized narratively. A total of 69 eligible studies evaluated 22 types of interventions: three universal, five selective, five indicated, and nine multi-level. Most studies were rated as high-quality (*n* = 61/69) and conducted in high-income countries (HICs) (*n* = 63/69), primarily assessing the cost-effectiveness of universal interventions like means restriction (*n* = 6), selective and indicated interventions like psychotherapy (*n* = 30), support services (*n* = 15), and medication (*n* = 5). Evidence consistently found that interventions for self-harm and suicide prevention were generally cost-effective or cost-saving. Strong evidence supported the cost-effectiveness of several universal (e.g. awareness training), selective (e.g. psychotherapy, support services), indicated (e.g. suicide risk screening, support services, psychotherapy for adults in HICs like Australia, US, Canada), and multi-level interventions. However, more economic evaluations are needed for interventions targeting older adults and children in all countries, especially in low- and middle-income countries, where evidence is lacking.

## Introduction

Self-harm and suicide (definitions in Table 1) have been recognized by the World Health Organization (WHO) as major public health concerns and consistently remained in the top four causes of death among individuals aged 15–29 year (WHO, 2021, 2023). These behaviors impose substantial economic burdens, with estimated average annual costs of $570 billion (2022 USD) in the US (Peterson, Haileyesus, & Stone, 2024) and $5.87 billion (2022 USD) in Australia (Kinchin & Doran, 2017), largely due to diminished quality-of-life, productivity losses, and healthcare expenditure. While comparable estimates are lacking for low- and middle-income countries (LMICs), the impact is likely substantial, with over 73% of global suicides occurring in LMICs in 2021 (WHO, 2023).

**Table 1.** Definitions of key terms Table 1. long description.

Term/Expression	Definitions
Self-harm	A non-fatal act where an individual causes harm to themselves, with various motives that may or may not involve the intention of ending their life but survives. A suicide attempt is a type of self-harm in which an individual harms themselves with the intention to die but survives (Pirkis et al., 2022).
Suicide	A fatal act that an individual initiated and carried out to deliberately end their own life, resulting in death (Pirkis et al., 2022).
Suicidal ideation	Thoughts of suicide that vary from a general sense of life being meaningless to an intense preoccupation with ending one’s own life (Pirkis et al., 2022).
Universal intervention	Target the entire population without determining who might be at risk of self-harm, suicide, or suicidal ideation (Pirkis et al., 2023).
Selective intervention	Focus on individuals displaying risk factors (e.g. stressful life events, family history of suicide, mental illness, impulsivity) that might lead to self-harm or suicide, even though they have not yet engaged in these behaviors (Pirkis et al., 2023).
Indicated intervention	Target individuals who have already engaged in self-harming or suicidal behaviors (e.g. people with previous self-harm episodes) (Pirkis et al., 2023).
Multi-level intervention	A combination of universal, selective, and indicated interventions delivered together (Hegerl, Heinz, O’Connor, & Reich, 2021).

A range of interventions (definitions in Table 1) have been shown to reduce self-harm and suicide, including universal interventions (e.g. restricting access to lethal means), selective interventions (e.g. medications like clozapine for people with mental illness), indicated interventions (e.g. psychological therapies for people with previous suicidal behaviors), and multi-level interventions (e.g. a combination of universal, selective, and indicated interventions) (Hegerl et al., 2021; Laflamme et al., 2022; Pirkis et al., 2024; Zalsman et al., 2016). However, evidence on the cost-effectiveness or value-for-money of these interventions still remains unclear, underscoring the need for reviewing economic evaluations to guide resource allocation (Kernick, 2003).

Earlier systematic reviews of economic evidence of self-harm and suicide preventive interventions tended to situate within interventions for mental illness and mental health promotion (Feldman et al., 2021; Ha et al., 2022; Le et al., 2021; Mihalopoulos & Chatterton, 2015; Mihalopoulos et al., 2011; Zechmeister et al., 2008). These reviews largely focused on interventions in high-income countries (HICs) and primarily covered educational and training interventions (Feldman et al., 2021; Mihalopoulos, Vos, Pirkis, & Carter, 2011; Zechmeister et al., 2008). To address these limitations, Madsen and colleagues (2018) conducted the first systematic review devoted exclusively to economic evaluations of self-harm and suicide interventions across both HICs and LMICs, identifying 25 studies. However, this review was conducted a decade ago, had limited coverage of studies published between 2003 and 2016, and likely underestimated return-on-investment (ROI) studies as ROI-related search terms were not included.

Since the review by Madsen and colleagues (2018), many economic evaluations of self-harm and suicide preventive interventions, including ROI studies, have been published, highlighting the need for an updated and more comprehensive synthesis. Expanding the coverage beyond the 2003–2016 period would enhance understanding of the interventions’ development history and assess whether interventions produce consistent economic outcomes across time and contexts (Drummond et al., 2009). With these motivations, this systematic review aims to provide a comprehensive and up-to-date overview of the available economic evidence on self-harm and suicide interventions without any restrictions (e.g. target population, prevention strategy) to identify those with strong cost-effectiveness potential.

## Methods

This systematic review conformed to the Preferred Reporting Items for Systematic Reviews and Meta-Analyses (PRISMA) guidelines (Page et al., 2021). The review protocol was registered in the PROSPERO database (#CRD42023460339).

### Identification and selection of studies

Eligible economic evaluations were identified through searches of multiple electronic databases, namely MEDLINE, Embase, PsycINFO, CINAHL, and EconLit, from inception to January 15, 2026. Reference list checks and grey literature searches on ProQuest and Google Scholar were also conducted. The search string was developed by adapting the search terms published in previous reviews (Madsen et al., 2018; Feldman et al., 2021; Le et al., 2021). Details on search strategies are provided in Supplementary Material 1.

Covidence was used to ensure that each record was independently screened by at least two reviewers at each stage of the review process. Four reviewers (AT, DL, DW, and PL) screened titles and abstracts, and two (DL, PL) reviewed the full text for appropriateness based on pre-defined inclusion and exclusion criteria (see below). High proportionate agreement was observed for both abstract (93.71%) and full-text (86.01%) reviews. A senior health economist (LL) arbitrated discrepancies among the four reviewers.

### Inclusion and exclusion criteria

The review included full economic evaluations assessing the cost-effectiveness of any preventive intervention for self-harm, suicide, and suicidal ideation, with no restrictions on prevention strategy, intervention type, target population, comparator, follow-up time, publication time, location, or setting. Eligible study designs included cost–benefit analysis (CBA), cost-minimization analysis (CMA), cost-utility analysis (CUA), cost-effectiveness analysis (CEA), or a ROI analysis that examined both the costs and benefits of at least two alternative courses of action (e.g. policies, interventions). The review excluded partial economic analyses (i.e. studies examined costs or benefits of both courses of action), studies focused on suicide gene therapy or accidental overdose, comments, editorials, opinion pieces, letters, conference abstracts, and studies written in languages other than English.

### Data extraction

Characteristics of the included studies were extracted and summarized in a table format adapted from prior systematic reviews (Le et al., 2021; Mihalopoulos & Chatterton, 2015). To ensure comparability, all costs were converted into 2022 US dollars using the purchasing power parity approach sourced from the International Monetary Fund – World Economic Outlook database (Shemilt, James, & Marcello, 2010). If the year of pricing was not specified, it was assumed to be 2 years prior to the year of publication. If the analysis perspective was not reported or unclear, it was inferred based on the cost and outcome measures. If the study did not calculate an incremental cost-effectiveness ratio (ICER), net monetary benefit (NMB), benefit-to-cost ratio (BCR), or ROI ratio, the extracted data on costs and outcomes were used to compute these ratios.

### Data synthesis

Interventions were first grouped into four strategies – universal, selective, indicated, and multi-level, and further classified by specific types like means restriction, medication, or psychological therapies (definitions in Supplementary Material 2). Given the substantial heterogeneity in intervention characteristics and economic evaluation methods, a meta-analysis was not conducted. Instead, a structured narrative synthesis was undertaken to evaluate and compare economic evidence across diverse interventions and contexts. Additionally, a dominance ranking framework derived from the Joanna Briggs Institute’s systematic review guidelines was employed to further categorize interventions through three color codes: red for ‘unfavored’ (higher costs and lower effectiveness), green for ‘favored’ (better outcomes at lower costs), and yellow for ‘unclear’ cost-effectiveness (e.g. more [less] effective and more [less] costly than the control) (Gomersall et al., 2015). For studies comparing multiple interventions against a control, each intervention’s cost-effectiveness was assessed separately. If conflicting cost-effectiveness outcomes appeared across different perspectives and/or outcome measures within a study, the intervention’s cost-effectiveness in that study was classified as unclear. If conflicting findings on cost-effectiveness were found among studies evaluating the same intervention type, the intervention type was concluded to have inconsistent results overall.

Although there is no consensus on cost-effectiveness thresholds for commonly used outcome measures such as costs per QALY gained or DALY averted (Pichon-Riviere et al., 2023; Sun et al., 2023), the threshold of 50,000 USD per QALY gained or DALY averted has been widely adopted (Neumann, Cohen, & Weinstein, 2014), aligning with the recommendations by decision makers in Australia (Productivity Commission, 2024) and in the UK (NICE, 2013). Accordingly, this review applied the above threshold to ensure consistency in cost-effectiveness judgments while also reporting the specific thresholds within the included studies in the data extraction tables. For studies employing less frequently used cost-effectiveness measures (i.e. cost per suicide case averted), judgments were based on the thresholds specified within each study.

### Methodology quality assessment

Drummond’s 10-item checklist was used to evaluate the methodological quality of the included studies (Drummond et al., 2015). Following prior publications (Madsen et al., 2018; Le et al., 2025), each item was scored based on whether it met (1 point), partially met (0.5 point), or did not meet (0 point) the criteria. A total score was used to classify study quality as high (8–10), average (5–7), or poor (0–4) (Le et al., 2025 5; Mihalopoulos et al., 2011). Two reviewers (PL and DL) assessed the quality of each study independently (proportionate agreement of 81.32%). Disagreements were resolved by discussion or arbitration by a third reviewer (LL).

## Results

**Figure 1.**
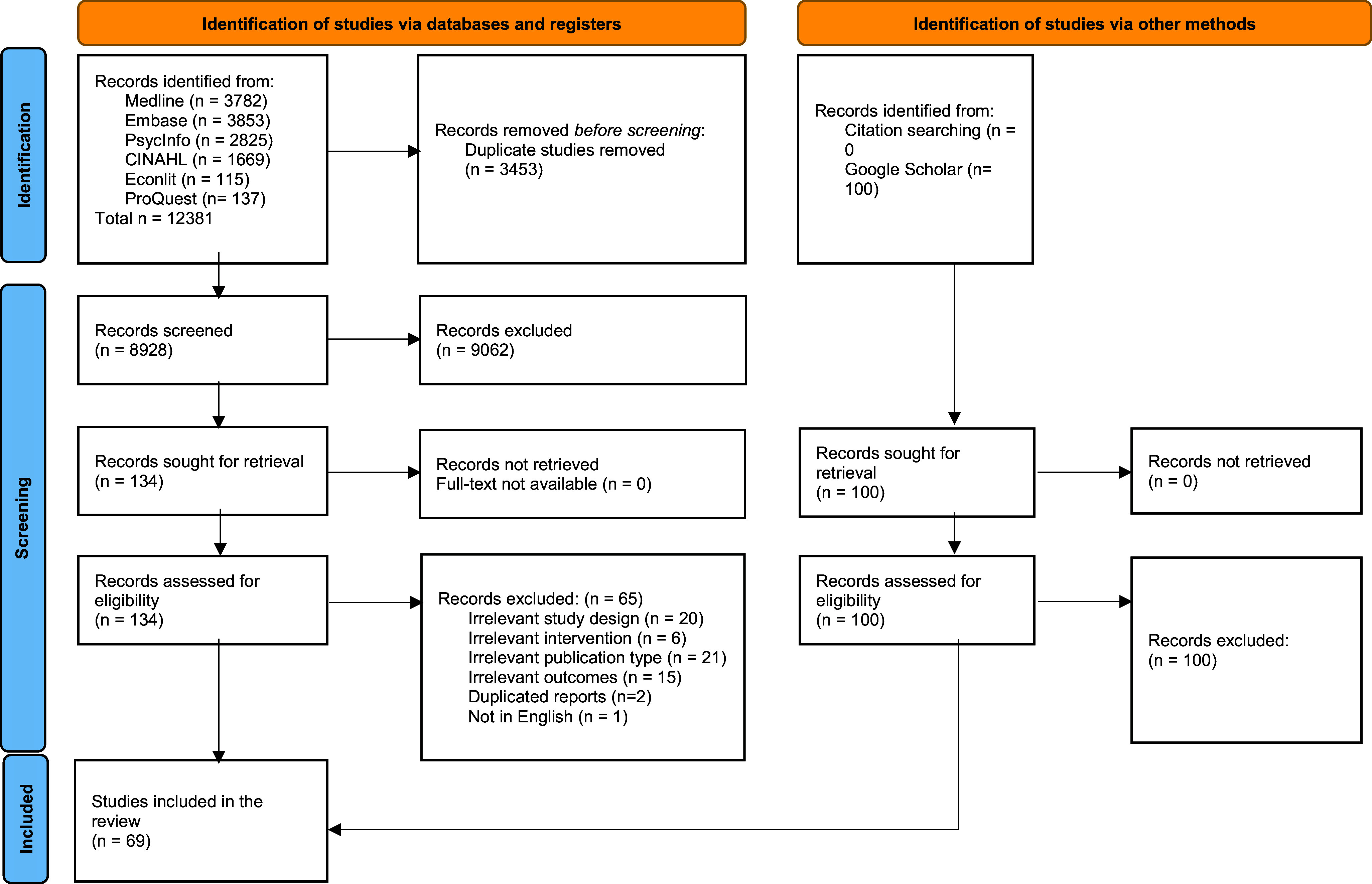
PRISMA flow diagram. Figure 1. long description.

The systematic searches identified a total of 12,381 records. After removing duplicates and screening titles, abstracts, and full-texts, 69 studies were eligible, including 30 studies identified in previous reviews (Madsen et al., 2018; Feldman et al., 2021; Ha et al., 2022; Le et al., 2021). No additional studies were identified through reference searches of included articles. Further details are presented in the PRISMA flow diagram (Figure 1).

### Characteristics of included studies

This review included 69 studies evaluating the cost-effectiveness of three types of universal, five types of selective, five types of indicated, and nine multi-level interventions for self-harm, suicide, and suicidal ideation prevention (Table 2). Most studies (*n* = 59) evaluated one type of intervention, with means restriction, support services, and psychotherapy were the most frequently evaluated universal, selective, and indicated interventions, respectively. Nearly all studies (*n* = 64) compared the interventions with no intervention or treatment-as-usual (TAU). Most studies were conducted in HICs, with only three from LMICs. The evaluation methods included CUA (*n* = 38), CEA (*n* = 37), ROI (*n* = 15), and CBA (*n* = 8). Over half of the studies (*n* = 39) were model-based with longer time horizons (typically 5 years to a lifetime), while the remaining were mostly trial-based with shorter time horizons (typically under 1 year). Only 19 studies targeted children and adolescents, and none focused on older adults.

**Table 2.** Characteristics of included studies Table 2. long description.

Lead author (year) country	Description of interventions	Description of comparators	Types of suicide targeted	Target population (age, %male)	Evaluation method	Study design	Perspective
1. Universal interventions							
Bandara (2022), Australia	Means restriction: Barriers installed at bridge and cliff sites	No Intervention	Suicide	People who attempt suicide at bridges and cliffs	ROI, CEA	Model (Decision tree)	Partial societal
Damerow (2020), Sri Lanka	Means restriction: Shop-based gatekeeper training program (n = 535 shops): delivered at local pesticide shops in 2-hour sessions by field staff members	No program	Suicide	People attempt suicide via pesticide poisoning	CEA	Model (Decision tree)	Government perspective
Law (2011), Hong Kong	Means restriction: Railway platform screen doors (PSDs) installation at the underground railway tracks	No intervention	Self-harm	General population	CUA	Quasi-experimental	Societal perspective
Lee (2021), Global (14 countries)	Means restriction: Banning highly hazardous pesticides	No intervention	Suicide	General population	CUA	Model (Markov)	Healthcare system
Whitmer (2013), US	Means restriction: Suicide barrier on the Golden Gate Bridge	No barrier	Suicide	People who attempt suicide at Golden Gate Bridge	CBA, CUA	Retrospective study	Partial societal perspective (assumed)
Jackson-Morris ( 2024), UK	Awareness training: Universal school-based training (5-h in-person sessions over 4 weeks by healthcare workers)	No intervention	Self-harm	All adolescents enrolled in a school (aged 10–19)	ROI, CUA	Model (Markov)	Partial societal perspective (assumed)
Kinchin (2020), Australia	Awareness training: Universal suicide prevention training (safeTALK) 3-hour workshop using presentations, videos, discussion, and questions	No intervention	Self-harm	Students aged 15–16 years	ROI	Model (Markov)	Health and justice system & Societal perspectives
Sari (2008), US	Awareness training: General suicide education: 5 lessons to provide college students with suicide facts, warning signs, and seeking help	No intervention	Suicide	Young adults aged 18–24 years	ROI	Model (Markov assumed)	Societal perspective
Flego (2022), Australia	Guidelines/Policies: Mindframe – Media guidelines for journalists as a universal suicide prevention strategy	No intervention	Suicide	General population	ROI, CEA, CUA	Model (Decision tree)	Societal perspective
2. Selective interventions							
Comans (2013), Australia	Support services: Standby Response Service: 24-hour multiple-center crisis response telephone number & in-person outreach (n = 90)	Usual care: (n = 670)	Suicide	People bereaved by suicide	CUA	Model (Markov)	Societal perspective
Pil (2013), Belgium	Support services: Chat helpline for suicide prevention Telephone helpline for suicide prevention	No intervention	Self-harm & Suicide	Unique users of the suicide helpline, who called/chatted about their personal problems	CUA	Model (Markov)	Societal perspective
Ross (2021), US	Support services: An active contact and follow-up (ACF) intervention	No intervention	Self-harm & Suicide	Primary care patients	CEA, CUA	Model (Decision analytic)	Healthcare system and Societal perspective
Sari (2008), US	Support services: Peer support group program: exploration of personal issues, build leadership & empathy, promote social skills	No intervention	Suicide	Young adults aged 18–24 years who may be at risk of suicide	ROI	Model (Markov assumed)	Societal perspective
Wilson-Barthes ( 2021), Kenya	Support services: Charitable Children’s Institutions: institutional care for orphanages	Family-based care	Suicidal ideation	Orphaned and separated children and adolescents (OSCA)	CEA, CUA	Prospective cohort study (*n* = 1,680)	Societal perspective
Gray ( 2011), US	Psychotherapy: Tailored and intensive family-centered in-home services (80–100 hours in 3 months); psychiatric treatment in mental health clinic (Pilot *n* = 22; At-scale *n* = 192)	TAU (Pilot *n* = 22, At-scale *n* = 611)	Suicidal ideation	Mentally ill teenagers (13–16 years old) at high risk for suicide within the juvenile court system	CEA	RCT for Pilot *n* = 44; Quasi-experimentation (At-scale *n* = 803)	Societal perspective (assumed)
Ross (2021), US	Psychotherapy: A cognitive behavioral therapy (CBT) intervention	No intervention	Self-harm & Suicide	Primary care patients	CEA, CUA	Model (Decision analytic)	Healthcare system and Societal perspective
Tebbett-Mock ( 2020), US	Psychotherapy: Dialectical behavior therapy (DBT): 9 weekly group training sessions for skill acquisition, daily intensive psychotherapy sessions in therapeutic groups (n = 425)	TAU	Self-harm	Adolescents (12–17 years) admitted to a coeducational, inpatient acute-care	CEA	Retrospective cohort study (n = 801)	Healthcare assumed
Zaloshnja (2003), US	Psychotherapy: Suicide prevention & intervention program: mental health technician support, 2 weekly psychologist sessions, monthly consultations with psychiatrist	No intervention	Suicide	Native American adolescents aged 15–19 years in communities with high risk of suicide	CUA, CBA	Model (Unclear)	Societal perspective
Dazelle (2026) the US	Medication: Oral iron therapy; Intravenous iron therapy (intravenous iron sucrose & ferric carboxymaltose)	Do nothing	Suicide	Pregnant women (M age = 28.5) with anemia	CEA, CUA	Model (Markov)	Healthcare & societal perspectives
Duggan (2003), UK	Medication: Clozapine	Conventional neuroleptic therapy	Suicide	Patients with severe mental illness & treatment-resistant schizophrenia	CEA, ROI	Model (Markov)	Healthcare system (NHS assumed)
Freemantle (1994), UK	Medication: Selective serotonin reuptake inhibitors or newer tricyclic and related antidepressants	Older tricyclics	Suicide	People with depression & take antidepressants	CEA	Model (Unclear)	Healthcare perspective assumed
Goren (2016), US	Medication: Clozapine: daily dose of 600 mg, monitoring (weekly for the first 6 month and biweekly for the next 6 months) (n = 18,000)	No clozapine	Suicide	Veterans with treatment-resistant schizophrenia	CEA	Model (Decision tree)	Healthcare provider (Veteran Health Admin)
Groessl (2018), US	Medication: Pharmacogenetic test (IDGx): buccal cell swabs with results used to guide medication selection	Standard of care (SOC)	Suicide	Patients with treatment-naïve or inadequately controlled major depressive disorder	CUA, CEA, CBA	Model (Markov)	Societal perspective
Hughes (2023), US	Health Policies: Psychologist prescriptive Authority: state policy granting prescriptive authority to qualified psychologists	No intervention	Self-harm & Suicide	People with mental health issues	CBA, CEA, CUA	Model (Markov)	Societal perspective
Park (2016), UK	Combined intervention: Early intervention: mental health services from 10 professionals over a year: medication, psychotherapy, counseling, vocational support.	Standard care	Suicide	People with first-episode psychosis	CEA	Model (Decision tree)	Broader economic (societal perspective assumed)
3. Indicated interventions							
Acolin (2022), US	Psychotherapy: Dialectical behavioral therapy (DBT): weekly individual & group sessions & clinical supervision for therapists for 1 year (n = 120)	Cognitive behavioral therapy (CBT): a brief group-based treatment (5–12 sessions) (*n* = 180)	Self-harm & Suicide	Adults aged 18–64 with previous suicide attempts	CEA, CUA	Model (Markov)	Healthcare system
Alvi (2022), Pakistan	Psychotherapy: Culturally adapted manual-assisted problem-solving training (C-MAP) plus TAU (*n* = 108): 6–8 sessions by a psychologist at home or healthcare setting	TAU (*n* = 113)	Suicidal ideation	Patients admitted after an episode of self-harm	CUA	RCT (*n* = 221)	Healthcare provider
Bernecker (2020), US	Psychotherapy: Brief cognitive behavioral therapy (BCBT): 12 individual sessions + TAU	TAU	Self-harm & Suicide	Soldiers with recent suicidal crises in the US Army	CEA	Model (Decision tree)	Program provider
Bjureberg (2025) Sweden	Psychotherapy: Internet-delivered emotion regulation individual therapy for adolescents (IERITA) + TAU: 11 digital modules for adolescents & 6 modules for parents over 12 weeks with support from a dedicated therapist	TAU: regular mental health services (therapy sessions biweekly)	Self-harm	Adolescents (M age = 15, 4% male) with self-harm in the past month	CEA, CUA	RCT (*n* = 166)	Healthcare & societal perspectives
Bojke (2024), UK	Psychotherapy: Family therapy: monthly 1.25-hour therapy session over 6 months	TAU	Self-harm	Youths (11–17 years) with self-harm at least twice	CUA	Model (Quasi-Markov)	Healthcare
Byford (1999), UK	Psychotherapy: Home-based social work intervention: assessment & 4 intensive, family-centered, home-based intervention sessions by psychiatric social workers + Routine care (*n* = 85)	Routine care (*n* = 77)	Suicidal ideation	Children aged <16 with deliberate self-poisoning	CEA	RCT (*n* = 162)	Societal perspective
Byford (2003), UK	Psychotherapy: Manual-assisted cognitive behavior therapy (MACT): treatment manual and up to 7 treatment sessions with a trained therapist (n = 239)	TAU (*n* = 241)	Self-harm	Patients aged 16–65 with a history of recurrent deliberate self-harm	CUA, CEA	RCT (*n* = 480)	Societal perspective (assumed)
Cottrell (2018), UK	Psychotherapy: Family therapy: monthly group supervision of 2 hours, monthly therapy session of ~1.25 hours over 6 months (n = 415)	TAU (*n* = 417)	Self-harm	Youths (11–17 years) with self-harm at least twice	CUA	Pragmatic, multi-center RCT (*n* = 832) + Model (Markov)	Healthcare system (NHS)
Denchev (2018), US	Psychotherapy: Suicide-focused (CBT) (9 sessions (bi)weekly psychotherapy).	TAU	Self-harm & Suicide	Adults with self-harm presenting to emergency care	CEA	Model (Markov)	Healthcare perspective assumed
Green (2011), UK	Psychotherapy: Group psychotherapy: 6 weekly psychotherapy sessions (acute phase) followed by a booster phase of weekly groups as needed + routine care (*n* = 183)	Routine care (*n* = 183)	Self-harm & Suicidal ideation	Adolescent (12–17 years) with at least 2 past episodes of self-harm in the previous year	CEA	RCT (*n* = 366)	Societal perspective
Haddock (2019), UK	Psychotherapy: Cognitive behavioral suicide prevention therapy: up to 20 sessions of 1 hour over 6 months in the community following discharge. + TAU (n = 24)	TAU (*n* = 27)	Suicidal ideation	In-patients (aged 18–65) in acute psychiatric wards with suicidal thoughts/behaviors	CUA	RCT (*n* = 51)	Healthcare (NHS), social care, and patient perspectives
Huntjens (2025), Netherlands	Psychotherapy: Dialectical behavioral therapy (DBT) (*n* = 63): weekly 45 min individual CBT, weekly 2-hour skill training group, telephone coaching, weekly 1-hour therapist consultation	CAU (*n* = 60): weekly 45-minute sessions with a psychotherapist or social worker	Suicide ideation	Autistic adults with suicidal behaviors (M age = 37.4, 52% male)	CEA, CUA	RCT (*n* = 123)	Healthcare & societal perspectives
Haga (2018), Norway	Psychotherapy: Dialectical behavioral therapy (DBT): 19 weekly 60-minute sessions of individual therapy, 120 min sessions of skills training, family therapy, telephone coaching, outpatient treatment (*n* = 39)	Enhanced usual care (EUC) (*n* = 38)	Self-harm	Adolescents aged 12–18 years with repeated self-harm, borderline personality disorder	CEA	RCT (*n* = 77)	Healthcare perspective
Krysinska (2024), Australia	Psychotherapy: Cognitive behavioral/problem-solving therapy	No intervention	Self-harm	Adults presented to hospitals with self-harm	ROI, CUA	Model (Markov)	Partial societal perspective
Lelie (2025), Belgium	Attempted Suicide Short Intervention Program (ASSIP): 3 sessions & one optional session after 3 week post discharge from hospital	CAU: short psychiatric evaluation, 3 outpatient sessions, 2 nights in psychiatric ward	Self-harm & Suicide	Patients admitted to hospital with previous suicide attempt (M age = unknown, 39% male)	CUA	Model (Markov)	Healthcare & societal perspectives
Martínez-Alés ( 2021), Spain	Psychotherapy: Problem-solving Psychotherapy program: an outpatient appointment, weekly individual psychotherapy over 2 months	TAU	Self-harm	Adults aged 18 years or above with previous suicide attempts	CEA	Model (Decision tree)	Societal perspective
Mavranezouli (2024), UK	Psychotherapy: (1) Cognitive behavioral therapy (CBT) + TAU: individual face-to-face psycho-education, problem solving (2) Dialectical behavioral therapy (DBT): weekly individual and group therapy sessions and support (consultation)	TAU: treatment provided by community mental health.	Self-harm	(1) Adults who have self-harm and present at hospitals (2) Children and young people who have self-harm and present at hospitals	CUA	Model (Decision analytic)	Healthcare perspectives
McCutchan (2022), US	Psychotherapy: The Collaborative Assessment and Management of Suicidality (CAMS): builds a therapeutic relationship and addresses suicidal thoughts through 4–11 structured weekly sessions	Enhanced treatment as usual (ETAU)	Suicidal ideation	Soldiers with current suicidal ideation	ROI	RCT (*n* = 148)	Healthcare system
Park (2018), Switzerland	Psychotherapy: Attempted Suicide Short Intervention Program (ASSIP): 3 60-minute face-to-face sessions, then semistructured personalized letters sent 6 times over 2 years	TAU	Self-harm	Individuals with suicide attempt and treated at hospital	CEA	RCT (n = 120)	Healthcare system
Priebe (2012), UK	Psychotherapy: Dialectical behavior therapy (DBT): weekly hour-long individual therapy sessions, 2-hour skills training group, and telephone coaching (*n* = 40)	Treatment as Usual (TAU) (*n* = 40)	Self-harm	Participants with personality disorder, ≥5 days of self-harm in the previous year	CEA	RCT (*n* = 80)	Societal perspective assumed
Tubeuf (2019), UK	Psychotherapy: Family therapy (FT): involved monthly sessions lasting 1.25 hour over 6 months, initially more frequent, totaling six to eight sessions. (*n* = 383)	Treatment as usual (TAU) (*n* = 371)	Self-harm	Adolescents aged 11–17 years with previous self-harm episode	CUA	RCT (*n* = 754)	Healthcare
Tyrer (2004), UK	Psychotherapy: Manual-assisted cognitive behavioral therapy (MACT): a booklet, 7 treatment sessions with a trained therapist (*n* = 239)	TAU (*n* = 241)	Self-harm	Individuals with a previous episode of self-harm and personality disorder	CEA	RCT (*n* = 480)	Broad economic (societal perspective)
Spijker (2012), Netherlands	Psychotherapy: Unguided Web-Based Self-help: six weekly modules on suicidal thoughts, intense emotions, negative thinking, and relapse prevention (*n* = 116)	Waitlist, a website that provided information on suicidality (*n* = 120)	Suicidal ideation	Adults (over 18 years old) with mild to moderate suicidal thoughts	CEA	RCT (*n* = 236)	Societal perspective
vanBentum (2025), Netherlands	Psychotherapy: Add-on module to reduce intrusive suicidal mental images + TAU (n = 46): 2–6 60-minute weekly sessions at mental healthcare center by trained therapists	CAU (*n* = 45): psychotherapy and/or pharmacotherapy	Suicide ideation	Adult psychiatric outpatients with depressive symptoms and distressing suicidal intrusions (M age = unknown, 32% male)	CUA	RCT (*n* = 91)	Healthcare & societal perspectives
Vasiliadis (2017), Canada	Psychotherapy: Increasing publicly funded access to psychotherapy: two visits with a GP, 8 sessions with a mental health professional	Status quo	Self-harm & Suicide	Adults aged 20–85 years with incident depression or self-harm	CUA, ROI	Discrete event simulation	Healthcare system and Societal perspective
Borschmann (2013), UK	Support services: Joint crisis plans (JCP) developed collaboratively and finalized with care coordinators, included in treatment plans alongside TAU to enhance crisis preparedness and support (*N* = 46)	Treatment as usual (*N* = 42)	Self-harm	Adults with borderline personality disorder & a self-harm record (Age M = 35.8, 19.3% male)	CUA	RCT (*N* = 88)	Health and social care sector perspective
Denchev (2018), US	Support services: (1) postcards/ letters (bi)monthly, (2) telephone outreach (1–3 months)	TAU	Self-harm & Suicide	Adults with self-harm presenting to ED	CEA	Model (Markov)	Healthcare perspective assumed
Gallien (2025), France	Support services: Brief contact intervention (BCI): post-hospital follow-up with structured crisis card, telephone calls, postcards	CAU	Self-harm & Suicide	Individuals admitted to hospital with suicide attempt (M age = 39, 37% male)	ROI, CBA	Retrospective cohort study (*N* = 2,3146)	Healthcare perspective (assumed)
Le (2023), Australia	Support services: Way Back Support Service: 4 brief aftercare interventions: (a) follow-up telephone contacts, (b) care coordination, (c) safety planning, (d) brief therapy	No intervention	Self-harm	Individuals aged 15+ years admitted to hospital/ emergency after a self-harm episode	ROI, CUA	Model (Markov)	Partial societal perspective
Lindkvist (2024), Sweden	Support services: Brief Admission by self-referral (BA) + TAU: up to 3 nights in the hospital, bring their own medication, supportive meeting with hospital staffs	TAU: access to outpatient and acute psychiatric clinics	Self-harm	Individuals with self-harm at risk of suicide (M age = 32, 12% Males)	CUA	RCT (*n* = 117)	Payer perspective
Martínez-Alés ( 2021), Spain	Support services: Enhanced Contact intervention: one-time priority outpatient appointment, in-person, and telephone follow-up	TAU	Self-harm	Adults aged 18 years or above with previous suicide attempts	CEA	Model (Decision tree)	Societal perspective
O’Connor (2017), UK	Support services: Volitional help sheet (VHS): a brief encouragement statement, a list of self-harm situations & potential solutions given within 24 hours after admission	TAU	Self-harm	Individuals aged 16+ years with self-reported self-harm and suicide attempt	CEA	RCT (*n* = 1,308)	Healthcare system
Richardson (2014), US	Support services: Post-discharge follow-up calls by eight call centers in collaboration with hospitals and emergency departments	No intervention	Self-harm & Suicidal ideation	In patients with previous self-harm or suicidal ideation	ROI	Model (Decision tree assumed)	Payer
Ryan (2015), the US	Support services: National Suicide Prevention Lifeline crisis center (telephone helpline): risk assessment, safety planning, referrals, emergency rescue activation	No intervention	Self-harm & Suicide	Callers with self-harm and suicide ideation (38% male)	CUA, CBA	Retrospective quasi-experimental study (*N* = 1,7202)	Societal perspective
Stelmach (2022), Global (36 countries)	Support services: Follow-up prevention in hospitals: patient education and follow-up	No intervention	Self-harm & Suicide	Adolescents aged 10–19 years with previous self-harm	CUA, ROI	Model (Markov)	Societal perspective
Appleby (2000), UK	Combined intervention: Suicide risk management training for professionals (2-hour sessions over 6 months) to provide assessment, clinical management, and problem-solving for patients at risk of suicide	No intervention	Suicide	Suicidal individuals in primary care, ED, mental health services	CEA	Pre-Post Study	Health and social care sector perspective
Dunlap (2019), US	Combined intervention: Screening by physician plus 7 telephone sessions, self-personal safety plan, treatment information, suicide hotline	TAU	Self-harm & Suicide	Individuals with self-harm ideation or behavior upon entry to emergency care	CEA	Cluster RCT (*n* = 1,376)	Program provider
Jackson-Morris ( 2024), UK	Combined intervention: a 1-hour information session at a hospital, 9 brief follow-up contacts over 18 months delivered by mental health support workers	No intervention	Self-harm	Adolescents with self-harm and treated at a hospital	ROI, CUA	Model (Markov)	Partial societal perspective (assumed)
Latimer (2014), Canada	Combined intervention: Rapid response team (RRT) (*n* = 158) included a psychiatrist, psychiatric nurse, and others offered assessment, treatment initiation (medication, CBT), and referrals	Usual care (*n* = 128)	Self-harm	Suicidal adolescents aged 12 to 17 years at emergency department	CEA	Trial (*n* = 286)	Healthcare & societal perspectives
Botchway (2022), UK	Screening: Oxford Mental Illness and Suicide (OxMIS): a suicide risk assessment for people with SMI (medication & clinical reviews, multi-disciplinary assessment, elective admission, ED visit)	Usual care	Suicide	People with severe mental illness (SMI) admitted to the hospital due to nonfatal self-harm	CUA	Model (Decision tree)	Healthcare system (NHS)
Dunlap (2019), US	Screening: Screening by ED nurses plus TAU	TAU	Self-harm & Suicide	Individuals with self-harm ideation or behavior upon entry to emergency care	CEA	Cluster RCT (*n* = 1,376)	Program provider
McDaid (2022), UK	Screening: Psychosocial assessment administered upon arrival to hospital due to self-harm	No intervention	Self-harm	Individuals admitted to hospital due to self-harm (age: 15+, 41%)	CUA	Model (Markov)	Healthcare and partial societal perspectives
de Beurs (2015), Netherlands	Training for professionals: e-learning program -Train-the-Trainer: 1 day in-person training with e-learning module for professionals + IAU (*n* = 312)	Usual implementation (IAU) (*n* = 254)	Suicidal ideation	Patients 18 and above with suicidal ideation	CUA, CEA	RCT (*n* = 566)	Societal perspective
4. Multi-level interventions							
Ahern (2018), Global (10 EU countries)	3-level School-based prevention: (1) Youth Aware of Mental Health (YAM): promote mental health knowledge (2) Question, Persuade, Refer (QPR): train gatekeepers to identify and intervene student’s risky behaviors. (3) Screening by Professionals (ProfScreen): screen, identify, and refer at-risk adolescents	Control group: only exposed to the same educational posters as in YAM	Self-harm & Suicidal ideation	School-age children and adolescents	CEA, CUA	Cluster-RCT (*n* = 11,110 students)	Payer (education, health, & social care system)
Crosland (2024), Australia	(1) Emergency department-based suicide prevention (EDSP): Universal and secondary suicide risk screening, discharge resources, follow-up phone calls	CAU	Self-harm and suicide	(1) Young people (aged 25 and below) with psychological distress	CUA, CEA, CBA	Model (Dynamic simulation techniques)	Healthcare and societal perspectives
Crosland (2024), Australia *(Continued)*	(2) School-based suicide prevention program (SSP): universal mental health education and awareness training; indicated brief screening and provision of CBT; treatment seeking assistance	CAU	Self-harm and suicide	(2) Children with psychological distress	CUA, CEA, CBA	Model (Dynamic simulation techniques)	Healthcare and societal perspectives
Doran (2016), Australia	Mates in Construction: awareness training (1 hr), connector (4 hr), intervention skills (2 days), promotion materials, 24/7 helpline	No program assumed	Self-harm & Suicide	Construction industry workers	ROI	Model (Decision tree)	Societal perspective (assumed)
Garraza (2018), US	Community-based suicide prevention programs (GLS): gatekeeper training, prevention hotlines, education, screening	No Intervention	Self-harm	Youths aged 16–23	ROI, CEA	Model (Unclear)	Healthcare system
Kinchin (2017), Australia	Mates in Construction: awareness training (1 h), connector (4 h), intervention skills (2 days), promotion materials, 24/7 helpline	No intervention	Self-harm & Suicide	Australian workforce (mean age = 42)	ROI	Model (Markov assumed)	Societal perspective
Lebenbaum (2020), Canada	Awareness campaigns, diagnostic training, educating primary-care physicians, & providing high-risk psychosocial interventions.	No intervention	Self-harm & Suicide	Ontario residents aged 16+ years (M age = 46 years)	CUA	Model (Markov)	Healthcare payer, Societal perspectives
Stelmach (2022), Global (36 countries)	Universal School-based prevention: gatekeeper training, mental health training for kids, screening, professionals’ referral (3 programs similar to Ahern)	No intervention	Self-harm & Suicide	Adolescents aged 10–19 years	CUA, CBA	Model (Markov)	Societal perspective
Vasiliadis (2015), Canada	Nuremberg Alliance Against Depression (NAD): community interventions for depression care and suicide prevention via universal awareness campaign, diagnostic and treatment training, and follow-up of individuals with previous self-harm	No intervention	Self-harm	First responders and follow-up of individuals who attempted suicide	CEA	Model (Markov assumed)	Healthcare system and Societal perspective

Lead author (year) country	Time horizon	Year of pricing, discount rates	Cost of intervention	Cost savings	Health outcomes/benefits	Results ICERS (in 2022 USD value) threshold	Quality
1. Universal interventions							
Bandara (2022), Australia	5 years 10 years	2018 USD, 3%	Initial installation and maintenance (cyclical and condition-based)	Value of statistical life	Suicide cases averted	ROI = 1.7 (95% UI, 1.0–1.9) (5 yr) and 2.4 (95% UI, 1.5 to 2.7) (10 yr) for building barriers at bridges. ROI = 1.7 (95% UI, −0.9–3.3, ns) (5 yrs), and ROI = 2.0 (95% UI, −1.1–3.8, ns) (for 10 yr) for building at cliff sites. ICER = dominant for barriers over bridges BUT not for cliff sites.	H
Damerow (2020), Sri Lanka	3 years	2019 USD, 3%	Direct personnel, travel, catering, admin, training materials, equipment, communication		Fatal pesticide self-poisoning case prevented; Life-year saved	Cost-effective if preventing 0.23 fatal pesticide self-poisoning cases over 3 years Threshold: $4,860/life-year saved	H
Law (2011), Hong Kong	25 years	2002 USD, 3%	Costs of installing railway platform screen doors	Passenger time Fare revenue saved after installing Railway platform screen doors	DALYs Number of suicidal and accidental falls	ICER = $122,000 per DALY averted and decreased to $103,000 if accounting for the loss of fare revenue and passengers’ waiting time Threshold: $117,000 per DALY averted	H
Lee (2021), Global (14 countries)	100 years	2017 I$, 3% for cost, 0% for health outcome	Program costs (admin, training, media, regulation) and patient-level costs	None	Healthy life-years gained (HLYGs) Suicide Mortality rate	ICER = $110 per HLYG (95% UI 86–145) in LMICs and $280 per HLYG (95% UI 225–356) in UMHICs Threshold: $118/HLYG	H
Whitmer (2013), US	20 years	2009 USD (assumed), unknown discount rate	Cost of implementing suicide barrier	Value of statistical life	Number of lives saved (Mortality reduction) DALYs	CBR = 1; ICER = $6,528 per DALY Threshold: $193,000/DALY averted	L
Jackson-Morris (2024), UK	10–80 years (lifetime)	2021 GBP, 3%	Salary, equipment (computer, phone call, internet access)	Long-term employment and earnings	DALYs	ICER = $4550 (10 years) & $970 (lifetime) /DALY averted ROIs = −0.4 (10 years) & 0.9 (lifetime)	H
Kinchin (2020), Australia	5 years	2017–18 AUD, 5% for costs	Travel and materials for local healthcare services and non-clinical groups.	Ambulance, healthcare, police, investigation, funeral, productivity losses, grieving	None	Health and justice perspective: ROI = 1.45 in Mackay; ROI = 0.19 in Queensland; ROI = 0.15 in Australia Societal perspective: ROI = 31.2 in Mackay; ROI = 4.1 in Queensland; ROI = 3.3 in Australia	H
Sari (2008), US	Lifetime (~40 years)	2000 USD, 6.3%	General suicide education: training & materials	Direct cost (ambulance & autopsy services); Indirect cost (earnings lost, productivity loss of family members)	None	ROI: 2.03	H
Flego (2022), Australia	5 years	2018 AUD, 3%	Staffing and non-staffing costs: review and update guidelines, training, support,	Costs of suicide death prevented (value of a statistical life, total cost associated with a suicide death)	Suicide deaths prevented QALY	ROI: 94 (95% CI 37–170) The intervention was dominant regarding cost per suicide prevented or QALYs gained Threshold: ROI > 1	H
2. Selective interventions							
Comans (2013), Australia	12 months 5 years	2010–2011 AUD assumed, 5%	Operating costs of providing intervention, community development, engagement, and training.	Health costs, Productivity lost (HPQ): Absenteeism, Presenteeism, unpaid work	QALY (EQ–5D)	The StandBy service is dominance WTP Threshold: $46,000/QALY gained	H
Pil (2013), Belgium	10 years	2012 EUR, 3% for cost and 1.5% for effects	Costs of salaries, transportation of personnel, stipends for volunteers, operation, and communication	Ambulance, medical examiner, ED, inpatient care, nursing home, productivity loss	QALYs (EQ–5D)	ICER = dominant Net societal savings of telephone and chat service: $3,600 (95%CI 2,960–4,330) and $3,460 (95%CI 2,800–4,200) in male users; $3,300 (95%CI 2,630–4,040) and $3,700 (95%CI 2,950–4,600) in female users.	H
Ross (2021), US	Lifetime	2016 USD, 3%	Intervention costs (No details)	Healthcare, patient time, productivity loss	Suicidal risk (Fatal and nonfatal suicide attempt) QALYs	To achieve ICERs below $50,000 per QALY gained, ACF needed a suicide prediction method with a sensitivity of ≥57% and specificity of ≥95%	H
Sari (2008), US	Lifetime (~40 years)	2000 USD, 6.3%	Peer support group: housing & food for retreat, trainers’ salaries, stipend, computer, transportation	Direct cost (ambulance services and autopsy services); Indirect cost (earnings lost & productivity loss of immediate family members)	None	ROI: 3.71	H
Wilson-Barthes (2021), Kenya	10 years	2018 USD, 3%	Personnel salaries, overhead, training, education fees, capital costs (e.g. appliances, equipment)	None	Reduction in suicidal ideation & intent (CDI-SF scale)	ICER for institutional care: $526 [179, 959] per unit reduction in suicidal diagnosis and $5,680 per QALY gained among OSCA WTP threshold: $8,100/QALY gained	H
Gray (2011), US	12 months	2009 USD assumed, no discount	Treatment costs & Cost of days spent in observation and assessment at detention centers and correctional facilities	Productivity loss of parents	Recidivism, days in court placement, Suicidal ideation (Youth Outcome questionnaire-YOQ)	Intervention is cost-saving in improving score on the youth outcome questionnaire for suicidal ideation	L
Ross (2021), US	Lifetime	2016 USD, 3%	Intervention costs (No details)	Healthcare, patient time, and lost productivity costs associated with fatal and non-fatal suicide attempts	Suicidal risk (Fatal and nonfatal suicide attempt) QALYs	To achieve ICERs below $50,000 per QALY gained, CBT needed a suicide prediction method with a sensitivity of ≥75% and specificity o ≥ 97%	H
Tebbett-Mock (2020), US	8 months	2017 USD assumed, no discount	None (Not provided)	Cost of constant observation hours	Incidents of suicide attempts, self-injury; restraints; and days hospitalized, aggression patient-to-patient/staff	Intervention is cost-saving: $141,000 for suicidal ideation, $90,000 for aggression, and $66,600 for self-injurious behaviors.	A
Zaloshnja (2003), US	Lifetime	1998 USD, 3%	Costs of salaries and educational	CUA: medical and out-of-pocket cost CBA: cost savings to individuals, local governments, insurance companies, employers, and the general public	QALYs	BCR: 43 ICER: $710 per QALY gained Threshold: $150,000/QALY gained	H
Dazelle (2026) the US	Lifetime	2020 USD, 3%	Costs of administering iron therapy	Medical costs (blood transfusions, premature births), productivity losses	QALY Adverse outcomes averted: post-hemorrhage, hysterectomies, suicide	ICER: dominant, Incremental NMB = $1,141–$2,128 Ferric carboxymaltose confers the greatest overall benefit Threshold: $100,000 per QALY gained	H
Duggan (2003), UK	10 years	2001 GBP assumed, 1.5% for outcomes & 6% for costs	Patient support costs Costs of clozapine & other alternative drugs	Costs of suicide: general hospital costs, inpatient costs	Life-years saved	Clozapine in treatment-resistant schizophrenia is cost-effective. Cost-neutral scenario: $12,000 per life-year saved Cost-saving scenario: $20,000,000 net annual saving	A
Freemantle (1994), UK	Lifetime	1992 GBP, 5%	Medication costs		Suicide cases & deaths related to anti-depressants (administrative data)	ICER = $54,600–$486,000 per life-year gained Selective serotonin reuptake inhibitors are more costly and more effective than the older tricyclic.	A
Goren (2016), US	12 months	2015 USD, no discount	Laboratory monitoring and follow-up visits, Medication costs	Direct healthcare cost: prescriptions, inpatient & outpatient treatment, emergency services, and office-based medical visits	Treatment response, suicides, adverse drug reactions & associated mortality, hospitalization due to clozapine	The intervention resulted in cost-saving per case of suicide-related death averted.	H
Groessl (2018), US	3 years	2016 USD, 3%	Cost of pharmacogenetic test	Direct medical costs (medications, hospitalizations, physician visits, psycho-therapy) Indirect medical costs (lost productivity)	QALYs (EQ–5D) Suicide rate	IDGx is dominant for moderate to severe MDD patients Threshold: $60,000 per QALY gained	H
Hughes (2023), US	20 years	2021 USD, 3%	Personnel, designation fee, maintenance, and individual prescribing psychologists	Medical costs (ED visits and hospitalization due to suicide) Societal costs (productivity, quality of life) following the suicide	Suicide attempt, suicide death, QALYs	ICER = $48,300 per QALY gained; $28,700 per nonfatal suicide attempt averted; 28,161 per suicide death averted Intervention is dominant at WTP threshold of $53,000	H
Park (2016), UK	10 years	2009 GBP, 3.5%	Costs of consultation with health professionals, formal and informal admission	Productivity losses, health service use, reduced quality of life	Suicide case averted	ICER = dominant (Societal cost savings of $1,815 per person per year)	H
3. Indicated interventions							
Acolin (2022), US	12 months	2022 USD, no discount	Cost of therapist’s time	Costs of hospitalization	Life years QALY	ICER = $26,362 per QALY gained ICERs for life-years gained or attempted suicides prevented were negligible. Threshold = $50,000 per QALY	H
Alvi (2022), Pakistan	6 months	2019 USD, no discount	Personnel travel and time, transportation and reimbursement, materials, supplies, and communication costs	Health services: Inpatient & outpatient, GP & other health professional visits	Suicidal ideation (Beck Scale for Suicidal ideation) QALY (EQ–5D–3L)	ICER for C-MAP = $18,410 (95% CI $8,060–$112,203) per QALY gained Threshold: no formal WTP threshold for Pakistan, thus used WTP threshold $22,600–$34,000	H
Bernecker (2020), US	Lifetime	2017 USD, Federal discount rate & 3%	Psychotherapy sessions, psychiatric inpatient days, & patients’ intake evaluations	Mortality costs, Suicide attempt costs, retired disability pay costs	Suicide attempts averted Suicide deaths averted	Cost saving = $17,700–$19,640/patient Except for the worst-case scenario, ICER of BCBT was dominant for $/suicide attempt & suicide death averted.	H
Bjureberg (2025) Sweden	1 & 3 months	2022 USD, no discount	Therapist’s time	Healthcare visits, medication, social support, productivity losses	Self-harm frequency (DSHI-Y), QALY (CHU–9D)	– ICERs - societal perspective: $18,677 (1 month) & $27,563 (3 month) per one adverted case; & $792,244 (1 month) & $471,800 (3 month) per QALY gained – ICERs – healthcare perspective: $15,502 (1 month) & $29,972 (3 month) per one adverted case; & $660,793 (1 month) & $361,041 (3 month) per QALY gained WTP threshold: $84,000 per QALY gained	H
Bojke (2024), UK	1.5 and 5 years	2022 GBP (assumed), 3.5%	Personnel costs, telephone contact, supervision	Costs of hospital admissions Non-hospital cost: primary & community care, medication	QALYs	ICERs = $53,000/QALY gained (1.5 years) – dominated (5 years) Threshold: $43,000/QALY gained	H
Byford (1999), UK	6 months	1997–1998 GPB, no discount	Costs of personnel, travel, supervision, hospital services, GP, social worker, and other healthcare and social services		Suicidal ideation (Suicidal Ideation Scale) Family functioning (Family Assessment Device) General Health	The intervention is as cost-effective as routine care alone For children with no depression, intervention is more effective in reducing suicidal ideation with no difference in costs.	H
Byford (2003), UK	12 months	1999/2000 GBP, no discount	Productivity losses, living expenses, hospital, community health, social services, criminal justice		Repeated episodes of deliberate self-harm (Parasuicide History Interview) HRQoL (EQ–5D)	At 12 months, MACT is dominant in reducing repeat self-harm, and ICER = $156,750/QALY gained. WTP Threshold: 0-$156,750/QALY gained	H
Cottrell (2018), UK	18 months 5 years	2014 GBP, 3.5%	Personnel costs, telephone contact, supervision	Healthcare costs (health, social services, in- & out-patient care) Medications	Hospitalization due to self-harm Suicidal ideation (BSS) QALY (PQ-LES-Q & GHQ–12)	At 18 months (base case), FT is dominated in preventing self-harm events & ICER = $64,185/QALY At 5 years, ICER = $34,000/QALY gained Threshold: $35,000–$52,000 per QALY gained	H
Denchev (2018), US	54 weeks	2014 USD assumed, no discount	Direct cost of intervention delivery	Costs of hospitalization & healthcare (ED visits, inpatient & outpatient care)	Self-harm and suicide incidents averted Life-years saved	ICER = $22,100 per life-year saved. WTP threshold: $61,000 per life-year saved	A
Green (2011), UK	12 months	2005–6 GBP, no discount	Salary of therapist, overhead, indirect therapist time (preparation), travel cost	Total costs of health, social care, education, and criminal justice sector services, plus family-related costs and productivity losses	Frequency of repeated self-harm (interview), severity of subsequent self-harm (triangulated reports), suicidal ideation (SIQ)	Group psychotherapy is not a cost-effective addition to the routine care delivered	H
Haddock (2019), UK	6 months	2015–16 GBP, no discount	Intervention cost	Community services, primary care, accident and emergency care, in- & out-patient care	Suicide behavior & ideation (SBQ, BSSI, BHS, clinical records); QoL (QoL-Brief); QALY (EQ–5D)	ICER = $637,000/QALY gained Intervention is less costly and less effective than TAU. 95CI suggests no difference in costs and QALYs for both groups	H
Huntjens (2025), Netherlands	12 months	2023 EUR, no discount	Costs of administering DBT & supervision costs for therapists providing DBT	Direct healthcare costs: health consultations, hospitalizations, medication Non-healthcare costs: carer costs, transportation, productivity losses.	QALY Averted suicidal ideation on SIDAS scale	ICER: dominant (healthcare perspective); & $1,527 per QALY gained & $1,642 per point improved on SIDAS scale (societal perspective) WTP threshold: $61,000 per QALY gained	H
Haga (2018), Norway	12 months	2012 EUR, no discount	Wages for staff, equipment, IT, house rent, telephone consultations, medication, therapist team consultations.	Health services due to self-harm (inpatient treatment, emergency care, and GP consultations	Self-harm episodes (LPC) Global functioning (CGAS)	ICER: less costly & more effective with 99.5% cost-effective at thresholds: $2,000/self-harm episode avoided	H
Krysinska (2024), Australia	10 years	2018 AUD, 3%	Costs of referral pathway, delivering intervention, weekly health professionals’ supervision cost	Costs of hospital admission related to self-harm, suicide, and death due to other causes	QALY	ROI = 2.3–6.0 ICERs = dominant Threshold: ROI > 1, $41,100 per QALY gained	H
Lelie (2025), Belgium	20 years	2023 EUR, 3% for costs, 1.5% for benefits	ASSIP start-up costs	Healthcare costs: psychiatric consultation, hospitalization Productivity loss: recuperation, and economic replacement costs	Suicide re-attempt or suicide death QALY	ICER: dominant (societal perspective), $360 per QALY gained (healthcare perspective) WTP threshold: $52,000 per QALY	H
Martínez-Alés (2021), Spain	12 months	2019 EUR, no discount	Costs of TAU plus 8 psychotherapy sessions.	Extra-hospital emergency care, in- & out-patient care. Indirect costs of death & disability leave	Suicide reattempt within 1 year after hospital discharge Time to reattempt	ICER = $10,600 per averted attempt WTP Threshold: $47,300–67,500/suicide averted	H
Mavranezouli (2024), UK	5 years	2020 GBP, 3.5%	(1) Costs of individual sessions (2) Costs of individual psychotherapy, group skill training, team and individual consultations	Healthcare costs associated with self-harm: primary care, emergency department, inpatient care, medication, social care	QALYs	(1) ICER = $13,800/QALY gained (2) ICER = $407,000/QALY gained Thresholds: $30,000–45,500 per QALY gained	H
McCutchan (2022), US	12 months	2018 USD, no discount	Implementation costs (training, personnel, travel, space, material, treatment costs (e.g. facilities, admin, insurance)	Healthcare expenditures, medical services, crisis services, & medications	Suicidal ideation (The Scale for Suicidal ideation-Current - SSI-C)	Mean CBRs at 12-month: −13.26 (SD = 55.10) for ETAU and 1.68 (SD = 40.53) for CAMS. Mean within-subject net benefit at 12-months: -$4,300 (SD = $17,000) for ETAU and -$480 (SD = $14,900) for CAMS.	H
Park (2018), Switzerland	24 months	2015 CHF, 3.5% for costs & 1.5% for health outcomes	Training costs for hospital staff	Inpatient care, outpatient care	Suicide attempt avoided	ASSIP was dominant, with significantly fewer reattempts at lower overall cost. WTP threshold: $0 and $25,600	H
Priebe (2012), UK	12 months	2010 GBP assumed, no discount	Service costs (no further details provided)	Costs of primary, secondary & social care: hospital, outpatient visits (GP), medication, lost workdays	Self-harm rate Borderline personality disorder	ICER = $67 per one percentage point reduction in the incidence of self-harm	H
Tubeuf (2019), UK	12 months	2017 GBP assumed, No discount	Costs of therapies sessions	Health service use (accident and ED visits and inpatient stays), psychotropic medications	QALYs (EuroQoL 5 Dimensions 3 Levels (EQ–5D–3L) for the adolescent and the Health Utility Index (HUI2) for one parent)	ICER = $67,500 per QALY gained (include adolescent’s health gain only) ICER = $45,400 - $68,200 per QALY gained (including adolescent’s health gain, health spill over to one parent) Threshold: $33,400–50,000/QALY gained	H
Tyrer (2004), UK	12 months	2002 GBP assumed, No discount	No details	Cost incurred by all service-providing sectors, productivity loss	Repetition in self-harm Time until repetition	No significant difference between self-harm cases in the MACT (39%) compared to TAU (46%) (P = 0.20), MAC was cost-effective (10% cheaper than TAU)	H
Spijker (2012), Netherlands	1.5 month (6 weeks)	2009 EUR, no discount	Costs of intervention, visits to psychologists, website maintenance, and hosting	Direct costs: medical (healthcare, prescription); non-medical (participants’ travel, loss of leisure time, informal care); Productivity losses	Change in suicidal ideation: Beck Scale for Suicidal ideation (BSS)/Suicidal ideation (BSS) Treatment response	ICER was cost saving = −$55,000 per additional treatment response. WTP Threshold: 0/treatment response	H
vanBentum (2025), Netherlands	12 months	2024 EUR, no discount assumed	Psychologists’ time providing add-on module	Healthcare costs: psychiatric healthcare, medication, out-of-pocket costs Productivity losses, travel expenses	QALY	ICER = $160,256 (societal perspective) & $116,605 (healthcare perspective) per QALY gained WTP threshold: $93,000 per QALY gained	H
Vasiliadis (2017), Canada	40 years	2012 CAD assumed, no discount specified	Costs of psychological therapy services for individuals received inadequate care	Medical, nonmedical, and productivity costs	QALYs	ICER = dominant (Societal cost savings: $2,700 per person (95% CI $1,340–$6,700)) ROI = 2.00 (1.78–3.15)	H
Borschmann (2013), UK	6 months	2009–2010 GBP, no discount	Costs of salaries, overheads, preparation & training	Costs of health and social care services: Hospital, community health and social care, medication, criminal justice sector services	Self-report self-harm post 6 months, Depression and anxiety (HADS), Well-being (WEMWBS), Perceived coercion (TES), QALY (EQ–5D)	JCP + TAU is dominant	H
Denchev (2018), US	54 weeks	2014 USD assumed, no discount	Direct cost of intervention delivery	Costs of hospitalization & healthcare (ED visits, inpatient & outpatient care)	Self-harm and suicide incidents averted Life-years saved	Postcards is dominant ICER = $5,280 (Telephone) per life-year saved. WTP threshold: $61,000 per life-year saved	A
Gallien (2025), France	12 months	2022 EUR, unspecified discount	Costs of administering BCI (unclear cost components)	Direct healthcare costs (assumed)	Suicide re-attempt or suicide death	ROI: 2.06 NMB per patient: $331 in healthcare cost saving	H
Le (2023), Australia	10 years	2018 AUD, 3%	Costs of salaries, staff training, professional development, and staff travel.	Healthcare cost-savings: (a) Base case: direct costs (healthcare, police & ambulance, & counseling), indirect costs (productivity loss); (b) SA: the value of a statistical life	QALYs	ROI = 1.58 (95% CI: −0.17 – 5.33) ICER = dominant (95% CI: dominant - $156,000/QALY) WTP Threshold: $41,000/QALY gained	H
Lindkvist (2024), Sweden	1 & 5 years	2020 EUR, 3%	Costs of brief admission: bed occupancy	Costs of psychiatric clinic visits, in-patient care, emergency department visits	QALYs	After 1 year: ICERs = cost-saving (capacity adjusted) - $79,000 (Base case) per QALY gained. All analyses indicated health gains and substantially reduced costs in 5 years.	H
Martínez-Alés (2021), Spain	12 months	2019 EUR, no discount	TAU costs plus service use costs (phone call, specialized visits).	Extra-hospital emergency care, in- & out-patient care. Indirect costs of death & disability leave	Suicide reattempt within 1 year after hospital discharge Time to reattempt	ICER = $4,000 per averted attempt WTP Threshold: $47,300–67,500/suicide averted	H
O’Connor (2017), UK	6 months	2014 GBP, no discount	Costs of health service use & routine implementation, excluding development costs (e.g. salaries)	Costs of hospital care, ambulance, treatment within EDs, hospital care following injury	self-harm event averted	VHS is dominant compared to TAU (lower costs and higher effective, although these differences are not statistically significant) WTP threshold: $0	H
Richardson (2014), US	1 month	2011 USD, no discount	Costs of follow-up calls, reports, admin work, and additional treatment	Mental health and substance abuse hospital readmissions	None	Patients discharged from (1) hospital: ROI = 1.76 (commercial insurance), 2.43 (Medicaid); (2) ED: ROI = 1.70 (commercial insurance), 2.05 (Medicaid)	H
Ryan (2015), the US	5 years	2013 USD, unspecified discount	Costs of staff, daily operations, facilities, technology, training	Emergency department costs, productivity losses	QALY	BCR = 0.27 ICER = $6,540 WTP threshold: $50,000 per QALY gained	H
Stelmach (2022), Global (36 countries)	80 years (Lifetime)	2019 USD, 3%	Salaries, medications, laboratory tests, computer equipment, material, transportation	Wage returns to education, productivity gains & losses	Utility weights converted into monetary unit DALY averted	ROI = 62 ICER = approximately <$110/DALY	H
Appleby (2000), UK	Lifetime	1998 GBP assumed, no discount	Material development, training, materials and equipment, and attending training		Suicide prevented: Life-year gained	ICER: $243,000 per suicide prevented and $8,300 per life-year gained.	L
Dunlap (2019), US	12 months	2015 USD, no discount	Wage & Salary, Rental costs for hospital space		Suicide attempts averted Suicide deaths averted	ICER = $6,100 per averted suicide attempt or death WTP threshold of $7,300	H
Jackson-Morris (2024), UK	10–80 years (lifetime)	2021 GBP, 3%	Salary, equipment (computer, phone call, internet access)	Long-term employment and earnings	DALYs	ICER = $10,900 (10 years) & $2,150 (lifetime) /DALY averted ROIs = 0 (10 years) & 2.9 (lifetime)	H
Latimer (2014), Canada	6 months	2011 CAD, no discount	Costs of RRT intervention and psychiatrist from RRT	Healthcare: ED visits, inpatient & outpatient care, medication Societal perspective: visits to other hospitals, out-of-pocket healthcare expenditures	Patient’s level of functioning (CGAS) Severity of suicidal behavior (SSBS)	Hospital perspective: ICER = $14,700 (dominant to $150,000) per point improved on the SSBS Societal perspective: ICER = $7,800 (dominant to $184,000) per point improved on the SSBS	H
Botchway (2022), UK	12 months	2021 GBP, no discount	Risk assessment administration, High risk management (e.g. additional 4 clinical reviews)	Direct costs of immediate emergency service and health-related.	QALYs	OxMIS is dominant	H
Dunlap (2019), US	12 months	2015 USD, no discount	Wage & Salary, Rental costs for hospital space		Suicide attempts averted Suicide deaths averted	ICER = $3,400 per averted suicide attempt or death WTP threshold of $7,300	H
McDaid (2022), UK	2 years	2020 GBP, 3.5%	Administering psychosocial assessment (No details)	Hospital costs of treating self-harm, ambulance costs, productivity loss	QALYs	ICERs = $16,600 (healthcare perspective); $15,100 (societal perspective) per QALY gained Thresholds: $30,000–45,500 per QALY gained	H
de Beurs (2015), Netherlands	3 months	2013 EUR assumed, no discount	Training costs & personnel costs	Direct medical costs (visits to a psychiatrist or a psychologist) Indirect medical costs (costs due to sick leave & productivity losses)	Suicidal ideation (BSS) QALYs (EQ–5D)	Intervention is not cost-effective compared to IAU (ICER = $3,900/point gained on BSS; ICER = $254,000/QALY gained) But cost-effective for suicidal patients if societal WTP > $14,000/point gained on BSS	H
4. Multi-level interventions							
Ahern (2018), Global (10 EU countries)	12 months	2010 EUR, no discount	Training, material adaptation, clinical interview, implementation, photocopy		Suicide attempt & Severe suicidal ideation (Paykel Hierarchical Suicide Ladder) QALY (SDQ)	ICERs per % reduction in suicide attempt = $134 (QPR), $52 (YAM), $78 (ProfScreen); ICERs per QALY gained = $179,187 (QPR), $69,876 (YAM), $95,191 (ProfScreen). WTP Threshold: $69,850/QALY gained ICERs per % point reduction in suicidal ideation = dominated (QPR), $68 (YAM), $152 (ProfScreen); ICERs per QALY gained = dominated (QPR), $71,660 (YAM), $161,684 (ProfScreen). WTP Threshold: $71,300/QALY gained YAM is the most cost-effective	H
Crosland (2024), Australia	10 & 30 years	2019–20 AUD, 5%	Intervention cost: program cost, administration cost	Healthcare, productivity, and carer costs	QALYs, Suicide deaths, and self-harm	(1) Healthcare perspective: ICER = $83,000 per QALY gained, $2,800 per self-harm prevented, $76,800 per suicide death prevented; INMB = −1; Societal perspective: ICER = $80,600 per QALY gained, $74,000 per suicide death prevented, $2,700 per self-harm prevented; INMB = −1; Thresholds: $63,700/QALY gained, INMB >0, no threshold for cost/suicide death or self-harm	H
Crosland (2024), Australia *(Continued)*	10 & 30 years	2019–20 AUD, 5%	Intervention cost: program cost, administration cost	Healthcare, productivity, and carer costs	QALYs, Suicide deaths, and self-harm	(2) Healthcare perspective: ICERs = dominated, $16.1 million per suicide death prevented, $0.22 million per self-harm prevented; INMB = −92; Societal perspective: ICER = dominated, $19.76 million per suicide death prevented, $0.27 million per self-harm prevented; INMB = −109; Thresholds: $63,700/QALY gained, INMB >0, no threshold for cost/suicide death or self-harm	H
Doran (2016), Australia	5 years	2010 AUD, 4.1%	No details on implementation cost	Production disturbance costs, human capital costs, medical costs, administrative costs, transfer costs, others	Potential suicide and self-harm cases averted (Administrative data)	ROI = 4.6	A
Garraza (2018), US	Lifetime	2010 USD, 3%	Staff salaries & benefits, travel, rent, phone, and other overhead expenses.	Avoided healthcare costs: inpatient stays, readmission, follow-up, transport, rehabilitation	Self-harm case avoided	ROI = 4.5 (95% CI: 1.59, 7.40) per dollar invested ICER = dominant	H
Kinchin (2017), Australia	Lifetime (~40 years)	2014 AUD, 4.58%	Average cost MIC per worker per year	Avoided production disturbance, human capital, medical, administrative, and other costs (carers, postvention services)	Suicide and self-harm cases averted	ROI = 1.50 (95% CI 1.11–3.07)	H
Lebenbaum (2020), Canada	Lifetime (50 years)	2016 CAD, 1.5%	Intervention cost per person (no further details)	Healthcare costs (hospitalization, ED, and family physicians), non-medical costs (autopsy, police & funeral services), productivity losses	QALYs (EQ–5D)	ICER = $19,300 per QALY (Societal perspective); $21,600/QALY (Healthcare payer perspective) Threshold: $51,000 per QALY gained	H
Stelmach (2022), Global (36 countries)	80 years (Lifetime)	2019 USD, 3%	Costs of salaries, salaries, medications, laboratory tests, computer equipment, materials, transportation	Wage returns to education, productivity gains & losses	Utility weights converted into monetary unit DALY averted	ROI = 3.5 ICER = approximately <$110/DALY	H
Vasiliadis (2015), Canada	Lifetime (~60 years)	2010 CAD, 3%	Cost of prevention program	Hospitalization & ED visits, outpatient medications, non-medical costs, police & funeral services, productivity losses & short-term disability	Life-years saved Suicide attempt case	NAD was cost-saving of $4,400 per life-year saved. ICER = $61,000 per case averted	H

*Note*: H, high quality; A, average quality; L, low quality.

### Summary findings

#### Universal interventions

***Universal means restriction:*** The cost-effectiveness of universal means restriction was inconsistent across four high-quality (Bandara et al., 2022; Damerow et al., 2020; Law & Yip, 2011; Lee et al., 2021) and one low-quality (Whitmer & Woods, 2013) studies. Barriers at bridges were cost-effective over 5–20 years from a partial societal perspective in Australia (Bandara et al., 2022) and the US (Whitmer & Woods, 2013) but not at cliff sites in Australia (Bandara et al., 2022) and railway platforms in Hong Kong (Law & Yip, 2011). Hazardous pesticide access restriction was cost-effective from a healthcare perspective across 14 countries over a lifetime (Lee et al., 2021). In Sri Lanka, a threshold analysis indicated that preventing as few as 0.23 suicides per 100,000 population over 3 years would render pesticide restriction cost-effective (Damerow et al., 2020).

***Universal awareness training:*** The cost-effectiveness of universal awareness training varied across three high-quality studies (Jackson-Morris et al., 2024; Kinchin et al., 2020; Sari et al., 2008). The intervention was cost-effective for self-harm prevention in Australia and in the US from a societal perspective (Kinchin et al., 2020; Sari et al., 2008). However, in the UK, Jackson-Morris et al. (2024) reported uncertain cost-effectiveness for self-harm prevention from a partial societal perspective.

***Universal media guidelines:*** In Australia, a high-quality study found media guidelines for reporting suicide were cost-effective in preventing self-harm and suicide in the general population over 5 years, compared to no intervention, from a societal perspective (Flego et al., 2022).

#### Selective interventions

***Selective support services:*** Support services were cost-effective in preventing self-harm, suicide, and suicidal ideation among individuals displaying risk factors (e.g. individuals facing life challenges) according to five high-quality studies (Comans, Visser, & Scuffham, 2013; Pil et al., 2013; Ross et al., 2021; Sari et al., 2008; Wilson-Barthes et al., 2021). In the US, active follow-up and peer support groups were cost-effective over a lifetime from healthcare and societal perspectives, respectively (Ross et al., 2021; Sari et al., 2008). Telephone and chat helplines also demonstrated cost savings from a societal perspective in Australia (Comans, Visser, & Scuffham, 2013) and Belgium (Pil et al., 2013). In Kenya, institutional care for orphaned children was more cost-effective than family-based care (Wilson-Barthes et al., 2021).

***Selective psychotherapy**:* The cost-effectiveness of selective psychotherapy for preventing self-harm, suicide, and suicidal ideation in populations displaying risk factors (e.g. mental illness) was demonstrated in four studies of mixed quality in the US (Gray et al., 2011; Ross et al., 2021; Tebbett-Mock et al., 2020; Zaloshnja et al., 2003). The intervention was cost-saving for adolescents at risk of mental illness over a 1-year period and a lifetime from healthcare and societal perspectives (Gray et al., 2011; Tebbett-Mock et al., 2020; Zaloshnja et al., 2003). However, among primary care patients, the intervention’s cost-effectiveness depended on whether suicide prediction scales had a minimum sensitivity of 75% and specificity of 97% (Ross et al., 2021).

***Selective medication**:* The cost-effectiveness of medication for populations displaying risk factors (e.g. mental illness, pregnant women with anemia) showed inconsistent results across five average-to-high quality studies (Dazelle et al., 2026; Duggan et al., 2003; Freemantle et al., 1994; Goren et al., 2016; Groessl et al., 2018). In the US, clozapine and antidepressants were cost-saving over 1–3 years from healthcare (Goren et al. 2016) and societal (Groessl et al., 2018) perspectives, respectively. Similarly, iron supplementation therapy was also cost-effective among pregnant women with anemia over a lifetime from healthcare and societal perspectives (Dazelle et al., 2026). However, in the UK, antidepressants (e.g. selective serotonin reuptake inhibitors) had uncertain cost-effectiveness, being more costly and more effective than older tricyclics, with ICERs of $54,600–$486,000 per life-year gained over a lifetime from a healthcare perspective (Freemantle et al., 1994). A threshold analysis over 10 years also showed a cost per life-year saved of $12,000 if clozapine were cost-neutral, and a cost saving if it reduced annual support costs by 10% from a healthcare perspective (Duggan et al., 2003).

***Selective health policies:*** A high-quality study in the US demonstrated that a health policy granting prescriptive authority for psychotropic medications to qualified psychologists was cost-effective in preventing self-harm and suicide among people with mental illness from a societal perspective over 20 years (Hughes et al., 2023).

***Selective combined intervention**:* Based on one high-quality study in the UK, the selective combined intervention (including medication, psychotherapy, counselling, and vocational therapies) was cost-saving compared to TAU for individuals with mental disorders over 10 years from a societal perspective (Park, McCrone, & Knapp, 2016).

#### Indicated interventions

***Indicated psychotherapy:*** Psychotherapy for individuals with previous self-harm episodes was largely cost-effective in preventing self-harm, suicide, and suicidal ideation across 25 studies, mostly high quality (*n* = 24) and conducted in HICs (*n* = 24).

Employing a 1-year time horizon, psychotherapy was found cost-effective for preventing self-harm, suicide, and suicidal ideation in adolescents and adults with previous self-harm across six studies, mostly of high-quality, undertaken in the US (Acolin, 2022; Denchev et al., 2018; McCutchan et al., 2022), Pakistan (Alvi et al., 2022), Norway (Haga et al., 2018), and Spain (Martínez-Alés et al., 2021). Specifically, this intervention was either cost-saving (McCutchan et al., 2022) or had acceptable ICERs (Alvi et al., 2022; Denchev et al., 2018; Haga et al., 2018; Martínez-Alés et al., 2021) compared to TAU from healthcare (Alvi et al., 2022; Denchev et al., 2018; Haga et al., 2018; McCutchan et al., 2022) and societal (Martínez-Alés et al., 2021) perspectives in these locations. In the US, dialectical behavior therapy was more cost-effective than cognitive behavior therapy from a healthcare perspective (Acolin, 2022).

However, mixed or unfavorable evidence was found in other locations and populations. Across seven high-quality studies in the UK, psychotherapy showed inconsistent cost-effectiveness for adults with previous self-harm (Byford et al., 2003; Haddock et al., 2019; Priebe et al., 2012; Tyrer et al., 2004) and was not cost-effective for adolescents with previous self-harm over 1 year or less (Byford et al., 1999; Green et al., 2011; Tubeuf, Saloniki, & Cottrell, 2019) from healthcare (Haddock et al., 2019; Tubeuf, Saloniki, & Cottrell, 2019) and societal perspectives (Byford et al., 1999, 2003; Green et al., 2011; Priebe et al., 2012; Tyrer et al., 2004). Similarly, three high-quality studies in the Netherlands reported inconsistent cost-effectiveness over a 1-year horizon or less from both healthcare and societal perspectives (Huntjens et al., 2025; Spijker et al., 2012; van Bentum et al., 2026). While psychotherapy demonstrated good value-for-money among adults with previous self-harm (Huntjens et al., 2025) or suicidal ideation (Spijker et al., 2012), it was not cost-effective among psychiatric outpatient adults experiencing suicidal intrusions (van Bentum et al., 2026). Over a shorter time horizon (1–3 months), a high-quality Swedish study found psychotherapy for adolescents with previous self-harm was not cost-effective (Bjureberg et al., 2025).

Over 2 years to a lifetime, the cost-effectiveness of psychotherapy for preventing self-harm and suicide among individuals with previous self-harm remained inconsistent across eight high-quality studies. In the US (Bernecker et al., 2020), Canada (Vasiliadis et al., 2017), Australia (Krysinska et al., 2024), and Switzerland (Park et al., 2018), psychotherapy was cost-saving compared to TAU or no intervention from healthcare (Park et al., 2018), payer (Bernecker et al., 2020), partial societal (Krysinska et al., 2024), and societal (Vasiliadis et al., 2017) perspectives. However, in the UK, the cost-effectiveness of psychotherapy compared with TAU was inconsistent across three studies (Bojke et al., 2024; Cottrell et al., 2018; Mavranezouli et al., 2024). Specifically, over a 5-year horizon, one study found psychotherapy cost-effective among adolescents (Cottrell et al., 2018), another demonstrated the intervention was dominated (Bojke et al., 2024), and a third found it cost-effective among adults but not among children (Mavranezouli et al., 2024), all from a healthcare perspective.

***Indicated support services:*** Support services were mostly cost-effective compared to TAU or no intervention in preventing self-harm, suicide, and suicidal ideation among individuals with previous self-harm, based on 10 studies of average (Denchev et al., 2018) to high (Borschmann et al., 2013; Gallien et al., 2025; Le et al., 2023; Lindkvist et al., 2024; Martínez-Alés et al., 2021; O’Connor et al., 2017; Richardson, Mark, & McKeon, 2014; Ryan, 2015; Stelmach et al., 2022) quality. Under 1-year time horizon, support services (e.g. postcard/telephone outreach) were cost-effective from payer, healthcare, and societal perspectives in the US (Denchev et al., 2018; Richardson, Mark, & McKeon, 2014), the UK (Borschmann et al., 2013; O’Connor et al., 2017), France (Gallien et al., 2025), and Spain (Martínez-Alés et al., 2021). However, findings from Sweden and the US showed unclear cost-effectiveness of support services, including brief hospital admission by self-referral (Lindkvist et al., 2024) and crisis helpline (Ryan, 2015) over 1 and 5 year(s), respectively. Over 10–80 years, support services (e.g. aftercare, follow-up contact) were dominant in Australia (Le et al., 2023) and across 36 HICs and LMICs (Stelmach et al., 2022) from societal and partial societal perspectives.

***Indicated suicide risk screening**:* Three high-quality studies from the US and the UK found suicide risk screening cost-effective for self-harm and suicide prevention among adults with previous self-harm or suicidal ideation in emergency departments from healthcare and societal perspectives over 1–2 year time horizon (Botchway et al., 2022; Dunlap et al., 2019; McDaid et al., 2022).

***Indicated training for professionals**:* A high-quality study found training for mental health professionals not cost-effective in preventing suicidal ideation among adults with prior suicidal ideation, but potentially cost-effective for those with depression and suicidal ideation compared to TAU over 3 months from a societal perspective in the Netherlands (de Beurs et al., 2015).

***Indicated combined interventions:*** The cost-effectiveness of indicated combined interventions for self-harm and suicide prevention among individuals with previous self-harm compared to TAU or no intervention remained inconsistent across one low-quality (Appleby et al., 2000) and three high-quality studies (Dunlap et al., 2019; Jackson-Morris et al., 2024; Latimer, Gariépy, & Greenfield, 2014) in HICs. In Canada, the combined intervention (assessment, referrals, and treatments) was less costly and less effective over 6 months from healthcare and societal perspectives (Latimer, Gariépy, & Greenfield, 2014). Conversely, in the US, the intervention (screening, safety/treatment plan, hotlines) was cost-effective for adults over 12 months from a program provider perspective (Dunlap et al., 2019). Nevertheless, in the UK, the cost-effectiveness of combined interventions (training professionals, follow-up contact, problem-solving for patients) remained uncertain from healthcare and partial societal perspectives over the lifetime (Appleby et al., 2000; Jackson-Morris et al., 2024).

#### Multi-level interventions

Multi-level interventions were mostly cost-effective in preventing self-harm and suicide over long time horizons (10 years to a lifetime) compared to no intervention, based on one average quality (Doran et al., 2016) and seven high-quality studies (Ahern et al., 2018; Crosland et al., 2024; Garraza et al., 2018; Kinchin & Doran, 2017; Lebenbaum et al., 2020; Stelmach et al., 2022; Vasiliadis et al., 2015).

In the US, a multi-level intervention (gatekeeper training, hotlines, education, screening) was cost-saving for youths (ages 16–23) with a ROI of 4.5 from a healthcare perspective (Garraza et al., 2018). However, similar interventions in Australia were not cost-effective for children and young people (aged below 25) with psychological distress from healthcare and societal perspectives (Crosland et al., 2024). Nevertheless, a multi-level intervention (awareness trainings, suicide intervention skills) for construction workers in Australia demonstrated positive ROIs of 1.5–4.6 across 5-year and lifetime horizons from a societal perspective (Doran et al., 2016; Kinchin & Doran, 2017). In Canada, two studies also found multi-level interventions (awareness campaigns, training, psychosocial interventions) to be cost-effective (Lebenbaum et al., 2020) or cost-saving (Vasiliadis et al., 2015) compared to no intervention over a lifetime from both healthcare and societal perspectives.

Globally, a study across 36 HICs and LMICs found a multi-level intervention (awareness campaigns, training, and screening) cost-effective among adolescents from a societal perspective over a lifetime (Stelmach et al., 2022). However, when components were examined separately in 10 European countries, their cost-effectiveness was uncertain from the education, health, and social care system perspective over 12 months (Ahern et al., 2018).

### Synthesis of findings

**Figure 2.**
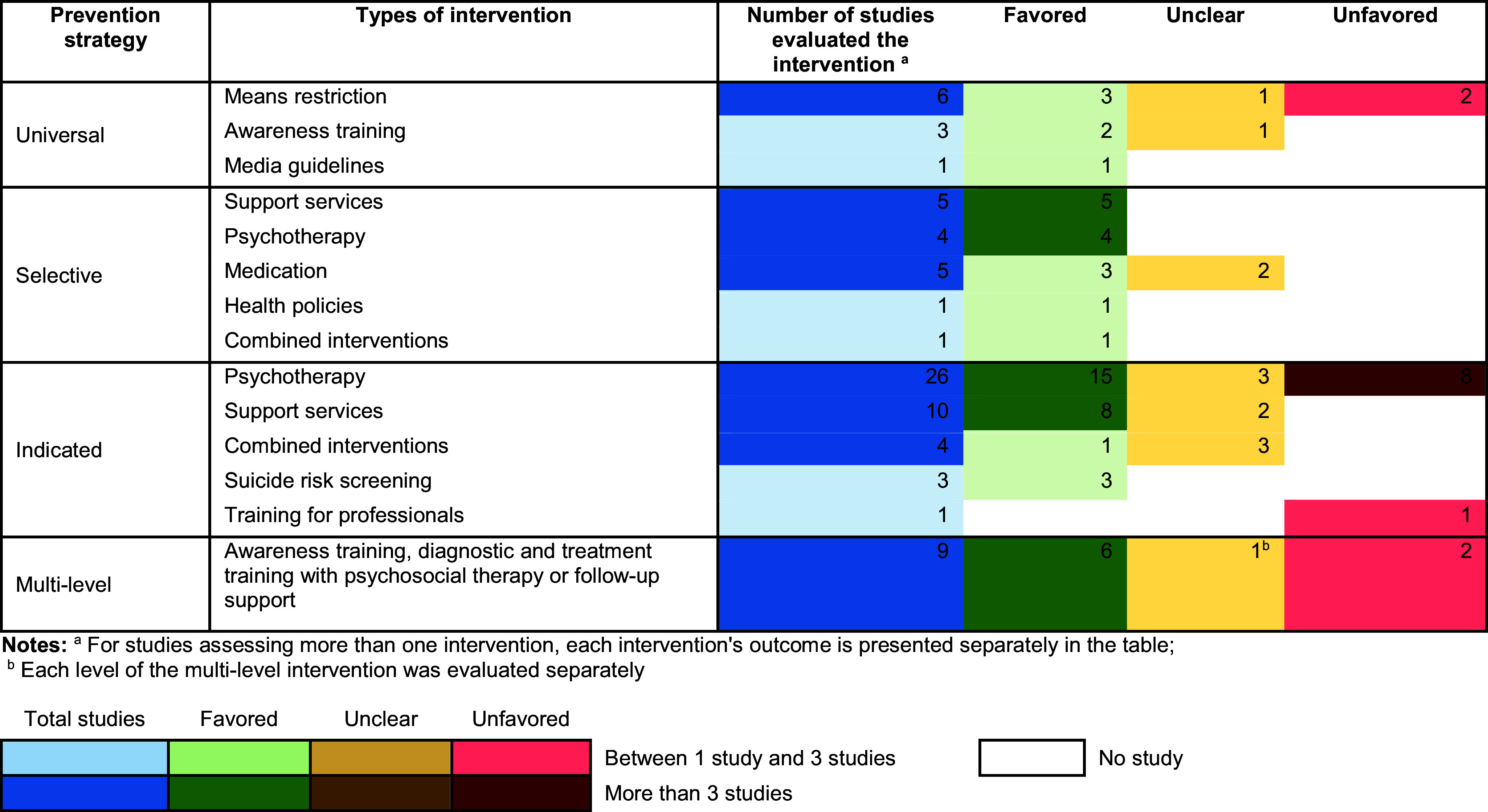
Cost-effectiveness results and implications for decision makers. Figure 2. long description.

As shown in Table 2, the included studies showed high heterogeneity in the intervention characteristics and economic evaluation methods, leading to the use of a dominance ranking framework with three classifications – ‘favored’, ‘unfavored’, and ‘unclear’ (Figure 2). The majority (67%) of studies reported ‘favored’ outcomes. While a few interventions (e.g. universal means restriction, indicated psychotherapy) demonstrated either ‘unclear’ or ‘unfavored’ results, several selective (e.g. psychotherapy, support services) and indicated (e.g. suicide risk screening) interventions consistently generated ‘favored’ cost-effectiveness. Notably, cost-effectiveness varied across countries and health system contexts, with several interventions appearing more consistently cost-effective in the US than in countries with universal healthcare systems (e.g. Australia, the UK), likely reflecting higher baseline healthcare costs and greater potential for cost offsets.

Although psychotherapy was the most frequently evaluated indicated intervention (*n* = 26), its cost-effectiveness remained inconsistent across the studies. Most studies (*n* = 15) reported ‘favored’ cost-effectiveness for psychotherapy in many countries (e.g. Australia, the US, Canada), though the results remained ‘unclear’ for adults and ‘unfavored’ for adolescents in the UK. In cases of ‘unclear’ cost-effectiveness, the primary reason was the absence of defined cost-effectiveness thresholds for outcome-specific measures (i.e. cost per suicide case averted).

Observations from interventions evaluated by multiple studies across three 10-year periods (1995–2025) revealed notable cost-effectiveness trends. Selective support services (Comans, Visser, & Scuffham, 2013; Pil et al., 2013; Ross et al., 2021; Sari et al., 2008) and selective psychotherapy (Gray et al., 2011; Ross et al., 2021; Zaloshnja et al., 2003) consistently demonstrated ‘favored’ cost-effectiveness across all periods. The cost-effectiveness of selective medication improved over time, shifting from ‘unclear’ in earlier periods (Duggan et al., 2003; Freemantle et al., 1994) to ‘favored’ in the most recent period (Goren et al., 2016; Groessl et al., 2018). Nevertheless, indicated psychotherapy exhibited inconsistent cost-effectiveness outcomes across multiple studies at different periods (Haddock et al., 2019; Spijker et al., 2012; Tubeuf, Saloniki, & Cottrell, 2019; Tyrer et al., 2004).

### Quality assessment

As shown in Table 3, most studies were of high quality (*n* = 61, 88%), with the majority of these (*n* = 47, 77%) published between 2015 and 2025, demonstrating an increase in publication quality and quantity over time. However, a few limitations persisted across several studies. Uncertainty and sensitivity analyses were often neglected or poorly conducted in many studies (e.g. Gray et al., 2011; Tebbett-Mock et al., 2020). Some studies inadequately addressed the generalizability of their findings and barriers to implementation (e.g. Tubeuf, Saloniki, & Cottrell, 2019; Whitmer & Woods, 2013). Additionally, the range of costs analyzed did not fully align with the stated perspective in certain studies (e.g. Appleby et al., 2000; Latimer, Gariépy, & Greenfield, 2014).

**Table 3.** Results of quality assessment using Drummond’s 10-item checklist Table 3. long description.

#	Lead author (year)	1. Was a well-defined question posed in answerable form?	2. Was a comprehensive description of the competing alternatives given?	3. Was the effectiveness of the program or services established?	4. Were all the important and relevant costs and consequences for each alternative identified?	5. Were costs and consequences measured accurately in appropriate physical units?	6. Were the costs and consequences valued credibly?	7. Were costs and consequences adjusted for differential timing?	8. Was an incremental analysis of costs and consequences of alternatives performed?	9. Was allowance made for uncertainty in the estimates of costs and consequences?	10. Did the presentation and discussion of study results include all issues of concern to users?	Total	Quality
1	Acolin (2022)	1	1	1	1	0.5	1	1	1	0.5	1	9	High quality
2	Ahern (2018)	1	1	1	1	1	1	1	1	1	1	10	High quality
3	Alvi (2022)	1	1	1	1	1	1	1	1	1	1	10	High quality
4	Appleby (2000)	1	0.5	0.5	0.5	0	0	0	1	0	1	4.5	Low quality
5	Bandara (2022)	1	1	1	1	1	1	1	1	1	1	10	High quality
6	Bernecker (2020)	1	1	1	1	1	1	1	1	1	1	10	High quality
7	Bjureberg (2025)	1	1	1	1	1	1	1	1	1	1	10	High quality
8	Bojke (2024)	1	1	1	1	1	0.5	1	1	1	1	9.5	High quality
9	Borschmann (2013)	1	1	1	1	1	1	1	1	0.5	0.5	9	High quality
10	Botchway (2022)	1	1	1	1	1	1	1	1	1	1	10	High quality
11	Byford (1999)	1	1	1	1	1	1	1	1	1	1	10	High quality
12	Byford (2003)	1	1	1	1	1	1	1	1	1	0.5	9.5	High quality
13	Comans (2013)	1	1	0.5	1	1	1	0.5	1	1	1	9	High quality
14	Cottrell (2018)	1	1	1	1	1	1	0.5	1	1	1	9.5	High quality
15	Crosland (2024)	1	1	1	1	1	1	1	1	1	1	10	High quality
16	Damerow (2020)	1	1	1	1	1	1	1	1	1	1	10	High quality
17	Dazelle (2026)	1	1	0.5	1	0.5	1	0.5	1	1	1	8.5	High quality
18	de Beurs (2015)	1	1	1	1	1	0.5	1	1	1	1	9.5	High quality
19	Denchev (2018)	0	1	1	0.5	1	0.5	1	1	1	0.5	7.5	Average quality
20	Doran (2016)	0.5	0.5	0.5	0.5	1	1	1	1	0.5	0.5	7	Average quality
21	Duggan (2003)	0.5	0.5	1	0.5	0	0	1	1	0.5	0.5	5.5	Average quality
22	Dunlap (2019)	1	1	1	1	1	1	1	1	0.5	1	9.5	High quality
23	Flego (2022)	1	1	1	1	1	1	0.5	1	1	1	9.5	High quality
24	Freemantle (1994)	0	0.5	1	0	1	1	0.5	1	0.5	1	6.5	Average quality
25	Gallien (2025)	0.5	1	1	0.5	0.5	1	1	1	1	1	8.5	High quality
26	Garraza (2018)	1	1	1	1	1	1	1	1	1	1	10	High quality
27	Goren (2016)	1	1	1	1	1	1	1	1	1	0.5	9.5	High quality
28	Gray (2011)	0	1	1	0.5	0	0	1	1	0	0.5	5	Low quality
29	Green (2011)	1	1	1	1	1	1	1	1	1	1	10	High quality
30	Groessl (2018)	1	1	1	1	1	1	1	1	0.5	1	9.5	High quality
31	Haddock (2019)	1	1	0.5	1	1	1	1	1	1	0.5	9	High quality
32	Haga (2018)	1	1	1	1	1	1	1	1	1	1	10	High quality
33	Hughes (2023)	1	1	1	1	1	1	1	1	1	1	10	High quality
34	Huntjens (2025)	1	1	1	1	1	1	1	1	1	1	10	High quality
35	Jackson-Morris ( 2024)	0.5	1	1	0.5	1	1	1	1	1	1	9	High quality
36	Kinchin (2017)	1	1	0.5	0.5	1	1	1	1	0.5	0.5	8	High quality
37	Kinchin (2020)	1	1	0.5	0.5	1	1	1	1	0.5	1	8.5	High quality
38	Krysinska (2024)	1	1	1	1	1	1	1	1	1	1	10	High quality
39	Latimer (2014)	1	1	1	0.5	1	1	1	1	1	0.5	9	High quality
40	Law (2011)	1	1	1	0.5	1	1	1	1	0	0.5	8	High quality
41	Le (2023)	1	1	1	1	1	1	0.5	1	1	1	9.5	High quality
42	Lebenbaum (2020)	1	1	1	1	1	1	1	1	1	1	10	High quality
43	Lee (2021)	1	1	1	1	1	1	1	1	1	1	10	High quality
44	Lelie (2025)	1	1	1	1	1	1	0.5	1	1	1	9.5	High quality
45	Lindkvist (2024)	1	1	1	1	1	1	1	1	1	1	10	High quality
46	Martínez-Alés ( 2021)	1	1	1	1	1	1	1	1	1	1	10	High quality
47	Mavranezouli (2024)	1	1	1	1	1	1	1	1	1	1	10	High quality
48	McCutchan (2022)	1	1	1	1	1	1	1	1	0.5	1	9.5	High quality
49	McDaid (2022)	1	1	1	1	1	1	1	1	1	1	10	High quality
50	O’Connor (2017)	1	1	1	1	1	1	1	1	1	1	10	High quality
51	Park (2016)	1	1	1	1	1	1	1	1	0.5	1	9.5	High quality
52	Park (2018)	1	1	1	1	1	1	1	1	1	1	10	High quality
53	Pil (2013)	1	1	0.5	1	1	1	1	1	1	0.5	9	High quality
54	Priebe (2012)	1	1	1	1	1	0.5	1	1	0.5	1	9	High quality
55	Richardson (2014)	1	1	1	0.5	1	1	1	1	1	0.5	9	High quality
56	Ross (2021)	1	1	1	1	1	1	1	1	1	1	10	High quality
57	Ryan (2015)	1	1	0.5	1	1	1	0	1	1	1	8.5	High quality
58	Sari (2008)	1	1	1	1	1	0.5	1	1	0.5	1	9	High quality
59	Stelmach (2022)	1	1	1	1	1	1	1	1	1	1	10	High quality
60	Tebbett-Mock ( 2020)	0	1	1	0	1	0.5	1	1	0	1	6.5	Average quality
61	Tubeuf (2019)	1	1	1	1	1	1	1	1	1	0	9	High quality
62	Tyrer (2004)	1	1	1	1	1	1	1	0.5	0.5	0	8	High quality
63	vanBentum (2025)	1	1	1	1	1	1	0.5	1	1	1	9.5	High quality
64	Spijker (2012)	1	1	1	1	1	1	1	1	1	1	10	High quality
65	Vasiliadis (2015)	1	1	1	1	1	1	0.5	1	0.5	1	9	High quality
66	Vasiliadis (2017)	1	1	0.5	1	1	0.5	0	1	1	1	8	High quality
67	Whitmer (2013)	0	1	0.5	0.5	0	0	0	1	0	0	3	Low quality
68	Wilson-Barthes ( 2021)	1	1	1	1	1	1	1	1	1	1	10	High quality
69	Zaloshnja (2003)	1	1	0.5	1	1	1	1	1	0	0.5	8	High quality

## Discussion

This review provides the most comprehensive analysis of existing economic evidence on self-harm and suicide prevention, covering a wide range of interventions and identifying significant growth in quality and quantity of publications, particularly in the past decade. Despite high heterogeneity, around 67% of the studies found suicide prevention to be cost-effective or cost-saving. Most cost-effectiveness evidence supported universal (e.g. awareness training), selective (e.g. support services), indicated (e.g. suicide risk screening, support services, psychotherapy for adults in Australia, the US, Canada), and multi-level interventions. However, our review also shows limited cost-effectiveness evidence of self-harm and suicide prevention in children, adolescents, and especially older adults, despite the high prevalence of such phenomena in these age groups (WHO, 2023). Similarly, there is significantly limited evidence from LMICs, which accounted for more than two-thirds of total suicides worldwide in 2021 (WHO, 2023).

### Policy implications and directions for future research

More economic evaluations are urgently needed, particularly in the underrepresented populations and regions mentioned above, to accelerate the implementation of cost-effective interventions. Critically, scaling up cost-effective interventions requires careful consideration of important local factors (Lee et al., 2021) because what works (or is cost-effective) in one context might not work (or be cost-effective) in another. For instance, system inefficiencies and higher baseline healthcare costs in the US may increase the potential for cost offsets from prevention, which may partly explain why preventive interventions often appear more cost-effective in US-based studies than in countries with universal healthcare. Furthermore, large-scale implementation requires a multidisciplinary approach impacting multiple sectors (WHO, 2021), which can lead to fragmentation of responsibility and funding across stakeholders.

### Methodological issues of current evidence

While most studies were high-quality, heterogeneity in outcome measures and comparators still exists, making direct comparisons and firm conclusions difficult across studies. Although suicide impacts broader society, many studies did not adopt a societal perspective, underestimating costs related to lost productivity, social services, and burden on families and communities (Werdin & Wyss, 2024). Additionally, inadequate discussion of broader implementation issues (e.g. generalizability and implementation barriers) and unclear cost-effectiveness thresholds, especially for clinical outcomes, might create hesitation and sub-optimal outcomes while implementing the interventions in the real-world.

### Strengths and limitations

To our knowledge, this is the first comprehensive and up-to-date review of the cost-effectiveness of self-harm and suicide interventions that employed broad inclusion criteria, including ROI studies. These ROI studies, accounting for 10% of the studies included, showed strong returns for self-harm and suicide interventions. However, the present review may have missed unpublished studies, studies published in grey literature (due to our limited search of grey literature), and studies written in non-English languages, which may underestimate the cost-effectiveness evidence from LMICs. In addition, the available evidence may be influenced by publication and commissioning practices. Nevertheless, the inclusion of studies reporting unfavorable or inconclusive cost-effectiveness findings suggests that negative economic results are not systematically excluded from the peer-reviewed literature.

## Conclusions

This review found strong and promising evidence for the cost-effectiveness of self-harm and suicide interventions, including several universal (e.g. awareness training), selective (e.g. psychotherapy, support services), indicated (e.g. suicide risk screening, support services, and psychotherapy for adults in countries including Australia, the US, Canada), and multi-level interventions. However, research on children, adolescents, and especially older adults remains limited, and evidence from LMICs, where most suicides occur, is also scarce. More economic evaluations for underrepresented populations and regions, as well as translational research on real-world implementation of suicide prevention, are urgently needed.

## Supporting information

10.1017/S0033291726104814.sm001 Le et al. supplementary material Le et al. supplementary material

## Supplementary material

The supplementary material for this article can be found at http://doi.org/10.1017/S0033291726104814.

## Table 1. Long description

The table has two columns. The left column lists terms: Self-harm, Suicide, Suicidal ideation, Universal intervention, Selective intervention, Indicated intervention, Multi-level intervention. The right column provides definitions. Self-harm is defined as a non-fatal act where an individual causes harm to themselves, with motives that may or may not include intent to die; suicide attempt is a type of self-harm with intent to die but survival. Suicide is a fatal act carried out to deliberately end one’s own life. Suicidal ideation refers to thoughts of suicide, ranging from a sense of meaninglessness to intense preoccupation with ending life. Universal intervention targets the entire population without assessing risk. Selective intervention focuses on individuals with risk factors such as stressful life events, family history, mental illness, or impulsivity, even if they have not engaged in self-harm or suicide. Indicated intervention targets those who have already engaged in self-harming or suicidal behaviors, such as people with previous self-harm episodes. Multi-level intervention combines universal, selective, and indicated interventions delivered together. Each definition cites Pirkis et al., 2022 or 2023, or Hegerl, Heinz, O’Connor, and Reich, 2021.

Navigate back to Table 1..

## Figure 1. Long description

Starting at the top left, the first track is labeled Identification of studies via databases and registers. Records identified from Medline 3782, Embase 3853, PsycInfo 2825, C I N A H L 1669, EconLit 115, ProQuest 137, total 12381. An arrow points right to Records removed before screening, Duplicate studies removed 3453. Downward, Records screened 8928, arrow to the right to Records excluded 9062. Downward, Records sought for retrieval 134, arrow right to Records not retrieved, Full-text not available 0. Downward, Records assessed for eligibility 134, arrow right to Records excluded 65, with breakdown: Irrelevant study design 20, Irrelevant intervention 6, Irrelevant publication type 21, Irrelevant outcomes 15, Duplicated reports 2, Not in English 1. Downward, Studies included in the review 69. The second track, right side, is labeled Identification of studies via other methods. Records identified from Citation searching 0, Google Scholar 100. Downward, Records sought for retrieval 100, arrow right to Records not retrieved 0. Downward, Records assessed for eligibility 100, arrow right to Records excluded 100. No studies from this track are included. All arrows indicate the flow of records through each phase, with exclusion points detailed at each step.

Navigate back to Figure 1..

## Table 2. Long description

This is a multi-column table with 15 columns and over 60 rows, organized by intervention type: Universal, Selective, Indicated, and Multi-level interventions. The first row contains headers: Lead author (year) country, Description of interventions, Description of comparators, Types of suicide targeted, Target population (age, percent male), Evaluation method, Study design, Perspective, Time horizon, Year of pricing and discount rates, Cost of intervention, Cost savings, Health outcomes/benefits, Results ICERS (in 2022 USD value) threshold, and Quality. Each subsequent row details a study, beginning with the lead author and year, followed by country, intervention description (e.g., means restriction, awareness training, support services, psychotherapy, medication, screening, policy), comparator (e.g., no intervention, treatment as usual), suicide type (e.g., suicide, self-harm, suicidal ideation), target population (with age and gender breakdowns), evaluation method (e.g., CEA, CUA, ROI, CBA), study design (e.g., model, RCT, cohort), perspective (e.g., societal, healthcare), time horizon (ranging from months to lifetime), year and currency for costs, intervention cost details, cost savings (direct and indirect), health outcomes (e.g., QALY, DALY, suicide cases averted), ICER or ROI results with thresholds, and study quality (H, A, L). Universal interventions include means restriction (barriers, gatekeeper training, banning pesticides), awareness training, and media guidelines. Selective interventions cover support services (helplines, peer support, institutional care), psychotherapy (CBT, DBT, family therapy), medication (clozapine, SSRIs, pharmacogenetic testing), and health policies. Indicated interventions focus on psychotherapy (DBT, CBT, family therapy, web-based self-help), support services (crisis plans, follow-up calls), and screening. Multi-level interventions include school-based, community, and workplace programs. Each study row provides specific data, such as sample size, cost components, outcome measures, and economic results (e.g., ROI values, ICERs per QALY or life-year gained, dominance statements). The table footnote defines quality ratings: H for high, A for average, L for low.

Navigate back to Table 2..

## Figure 2. Long description

The table has four main rows for prevention strategy: Universal, Selective, Indicated, and Multi-level. Each row lists types of intervention in the second column. The third column shows the number of studies evaluating each intervention, with blue for 1 to 3 studies and dark blue for more than 3. The next three columns show study outcomes: Favored (green), Unclear (yellow), and Unfavored (red). Universal strategies include means restriction (6 studies: 3 favored, 1 unclear, 2 unfavored), awareness training (3 studies: 2 favored, 1 unclear), and media guidelines (1 study: 1 favored). Selective strategies include support services (5 studies: 5 favored), psychotherapy (4 studies: 4 favored), medication (5 studies: 3 favored, 2 unclear), health policies (1 study: 1 favored), and combined interventions (1 study: 1 favored). Indicated strategies include psychotherapy (26 studies: 15 favored, 3 unclear, 8 unfavored), support services (10 studies: 8 favored, 2 unclear), combined interventions (4 studies: 1 favored, 3 unfavored), suicide risk screening (3 studies: 3 favored), and training for professionals (1 study: 1 unfavored). Multi-level strategies (awareness training, diagnostic and treatment training with psychosocial therapy or follow-up support) have 9 studies: 6 favored, 1 unclear, 2 unfavored. The legend at the bottom explains color codes and study count ranges. Notes clarify that multi-level interventions are evaluated separately and that each intervention’s outcome is presented individually.

Navigate back to Figure 2..

## Table 3. Long description

The table contains 69 rows, each representing a study, and 14 columns. The columns are: number, lead author with year, ten checklist items (each scored 0, 0.5, or 1), total score, and quality rating. The checklist items are: 1. Was a well-defined question posed in answerable form 2. Was a comprehensive description of the competing alternatives given 3. Was the effectiveness of the program or services established 4. Were all the important and relevant costs and consequences for each alternative identified 5. Were costs and consequences measured accurately in appropriate physical units 6. Were the costs and consequences valued credibly 7. Were costs and consequences adjusted for differential timing 8. Was an incremental analysis of costs and consequences of alternatives performed 9. Was allowance made for uncertainty in the estimates of costs and consequences 10. Did the presentation and discussion of study results include all issues of concern to users. For each study, the checklist items are scored, summed for a total (maximum 10), and assigned a quality rating: High quality, Average quality, or Low quality. Most studies score between 7 and 10 and are rated High quality. A few studies, such as Whitmer and Gray, have lower totals (3 and 5) and are rated Low quality. The table allows comparison of methodological quality across studies.

Navigate back to Table 3..
