# Supplementary material for: Economic evaluations of preventive interventions for self-harm and suicide: a systematic review
Source: Psychol Med. 2026 Jun 17;56:e195. doi: 10.1017/S0033291726104814 (PMC13280699; doi:10.1017/S0033291726104814)
Supplement: Le et al. supplementary material [file S0033291726104814sup001.docx]

**Supplementary Materials**

**Supplementary 1: Search terms**

1. Search terms for MEDLINE, Embase, PsycINFO, CINAHL, and EconLit

| **Concept 1:**  Health Economics | Keywords/Synonyms: | cost* **OR** efficien* **OR** cost-effect* **OR** cost effective* **OR** cost sav* **OR** cost offset* **OR** cost utilit* **OR** cost-analys* **OR** cost-minimi?ation analys* **OR** cost minimi?ation analys* **OR** cost adj1 benefit **OR** cost-benefit analys* **OR** cost benefit analys* **OR** cost-utility analys* **OR** cost utility analys* **OR** cost-effective analys* **OR** cost effective analys* **OR** cost-consequence analys* **OR** cost consequence analys* **OR** cost analys* **OR** cost per death averted **OR** cost per disability-adjusted life year averted **OR** cost per DALY averted **OR** economic evaluation* **OR** economic-effective* **OR** economic efficien* **OR** value for money **OR** return o? investment **OR** return to investment **OR** social return o? investment **OR** social return to investment **OR** return on |
| --- | --- | --- |
|  | Subject headings/MeSH: | exp "costs and cost analysis"/ **OR** exp "cost allocation"/ **OR** exp cost-benefit analysis/ **OR** exp "cost control"/ **OR** exp "cost of illness"/ **OR** exp cost-effectiveness analysis/ |
| **Concept 2:**  Phenomenon | Keywords/Synonyms: | suicidal* **OR** suicid* **OR** self-harm* **OR** selfharm* **OR** (self AND harm*)  **OR** selfinjur* **OR** self-injur* **OR** (self AND injur*) **OR** selfpoison* **OR** self-poison* **OR** (self AND poison*) **OR** selfcut* **OR** self-cut* **OR** (self AND cut*) **OR** parasuicid* **OR** para-suicid* **OR** ((deliberat* or intent*) AND overdos*) **OR** Grie* **OR** Mourn* **OR** Bereav* **OR** Suicide bereave* **OR** Bereave* by suicide **OR** loss by suicide **OR** suicide loss survivor* |
|  | Subject headings/MeSH: | exp Suicide/ **OR** exp Suicide, Attempted/ **OR** exp Self-Injurious Behavior/ **OR** exp bereavement/ **OR** exp Grief/ **OR** exp Prolonged Grief Disorder/ **OR** exp “Drug Overdose”/ |
| **Concept 3:**  Prevention/  Intervention/  Promotion | Keywords/Synonyms: | Prevent* **OR** Promot* **OR** intervent* **OR** control* **OR** manage* **OR** treat* **OR** reduc* **OR** stop* **OR** restrain* **OR** trial* **OR** universal prevention **OR** selective prevention **OR** indicated prevention **OR** primary prevention **OR** secondary prevention **OR** tertiary prevention **OR** postvention |
|  | Subject headings/MeSH: | exp Health Promotion/ **OR** exp Health Knowledge, Attitudes, Practice/ **OR** (Accident Prevention **OR** Prevention, Accident **OR** Prevention, Post-Exposure **OR** Prevention, Primary **OR** Prevention, Secondary **OR** Prevention, Suicide **OR** Primary Prevention **OR** Secondary Prevention **OR** Suicide Prevention).mp. |

1. Search terms for Google Scholar

(“suicide” OR “self-harm” OR "suicidal ideation") AND (“prevention” OR “intervention” OR “programme” OR “program”) AND ("cost-effectiveness" OR "cost effectiveness" OR "economic evaluation" OR "cost-benefit" OR "cost utility" OR "cost analysis" OR "return on investment" OR “ROI”)

1. Search terms for ProQuest

(“suicide” OR “self-harm” OR "suicidal ideation") AND (“prevention” OR “intervention” OR “programme” OR “program”) AND (“cost-effectiveness” OR “cost effectiveness” OR “economic evaluation” OR “cost-benefit” OR “cost utility” OR “cost analysis” OR "return on investment" OR “ROI”)”

**Supplementary 2: Definition of intervention types**

| **Intervention type** | **Definition** |
| --- | --- |
| Means restriction | Interventions that limit access to highly lethal methods for suicide (e.g. pesticides, firearms, medications, jumping sites) in order to reduce suicide mortality (Sultan et al., 2021; Yip et al., 2012) |
| Awareness training | Educational or community‑based programmes designed to increase recognition of suicide warning signs, reduce stigma, and promote help‑seeking, often delivered to the general public or specific groups (e.g. students, workplaces) (Goldsmith et al., 2002) |
| Media guidelines | Guidelines that promote responsible reporting and messaging about suicide, aiming to reduce suicide contagion and encourage help‑seeking by avoiding sensationalism and method description (World Health Organization, 2023) |
| Support services | Non‑clinical or semi‑clinical services that provide crisis support, outreach, or linkage to care, such as crisis hotlines, peer support, community crisis teams, and post‑attempt follow‑up services (Sultan et al., 2021) |
| Psychotherapy | Structured, evidence‑based psychological treatments that directly target suicidal thoughts and behaviours, such as cognitive‑behavioural therapy, dialectical behaviour therapy, or suicide‑specific psychotherapies (Calati et al., 2022) |
| Medication | Pharmacological interventions used to reduce suicide risk, either by treating underlying psychiatric disorders or via agents with specific anti‑suicidal effects (e.g. lithium, clozapine) (Zisook et al., 2023) |
| Health policies | Legislative, regulatory, or system‑level actions that shape environments, services, or access to care relevant to suicide prevention (e.g. national strategies, insurance coverage) (Pirkis et al., 2024) |
| Suicide risk screening | The use of standardised tools or protocols to identify individuals at elevated risk of suicide, typically in healthcare or community settings, followed by referral or assessment (National Institute of Mental Health, 2024) |
| Training for professionals | Structured training programmes for healthcare, social service, or community professionals to identify, assess, and respond to suicide risk, including gatekeeper and clinical training (Sultan et al., 2021) |
| Combined intervention | Interventions that comprise several intervention components (i.e. awareness training, psychotherapy) within the same prevention strategy (i.e. universal, selective, or indicated). |

**References**

Calati, R., Mansi, W., Rignanese, M., Di Pierro, R., Lopez-Castroman, J., Madeddu, F., & Courtet, P. (2022). Psychotherapy for Suicide Prevention. In *Suicide Risk Assessment and Prevention* (pp. 1173–1206). Springer, Cham. https://doi.org/10.1007/978-3-030-42003-1_70

Goldsmith, S. K., Pellmar, T. C., Kleinman, A. M., & Bunney, W. E. (2002). Programs for Suicide Prevention. In *Reducing Suicide: A National Imperative*. National Academies Press (US). https://www.ncbi.nlm.nih.gov/books/NBK220931/

National Institute of Mental Health. (2024). *Ask Suicide-Screening Questions (ASQ) Toolkit*. https://www.nimh.nih.gov/research/research-conducted-at-nimh/asq-toolkit-materials

Pirkis, J., Dandona, R., Silverman, M., Khan, M., & Hawton, K. (2024). Preventing suicide: A public health approach to a global problem. *The Lancet Public Health*, *9*(10), e787–e795. https://doi.org/10.1016/S2468-2667(24)00149-X

Sultan, S., Linskens, E., Gustavson, A., Sayer, N., Murdoch, M., MacDonald, R., McKenzie, L., Ullman, K., Venables, N., & Wilt, T. J. (2021). *Systematic Review: Population and Community-based Interventions to Prevent Suicide*. Department of Veterans Affairs (US). http://www.ncbi.nlm.nih.gov/books/NBK585079/

World Health Organization. (2023). *Preventing suicide: A resource for media professionals, update 2023*. https://www.who.int/publications/i/item/9789240076846

Yip, P. S., Caine, E., Yousuf, S., Chang, S.-S., Wu, K. C.-C., & Chen, Y.-Y. (2012). Means restriction for suicide prevention. *The Lancet*, *379*(9834), 2393–2399. https://doi.org/10.1016/S0140-6736(12)60521-2

Zisook, S., Domingues, I., & Compton, J. (2023). Pharmacologic Approaches to Suicide Prevention. *Focus*, *21*(2), 137–144. https://doi.org/10.1176/appi.focus.20220076
